# Gigaxonin E3 ligase governs ATG16L1 turnover to control autophagosome production

**DOI:** 10.1038/s41467-019-08331-w

**Published:** 2019-02-15

**Authors:** Aurora Scrivo, Patrice Codogno, Pascale Bomont

**Affiliations:** 1 Avenir-Atip team, INM, INSERM, Université Montpellier, 34091 Montpellier, France; 2 Institut Necker-Enfants Malades (INEM), INSERM U1151-CNRS UMR8253, 75993 Paris, France; 3 Université Paris Descartes-Sorbonne Paris Cité, 75006 Paris, France; 4 Present Address: Department of Developmental and Molecular Biology, Albert Einstein College of Medicine, 10461 New York, NY USA

## Abstract

Autophagy is an essential self-digestion machinery for cell survival and homoeostasis. Membrane elongation is fundamental, as it drives the formation of the double-membrane vesicles that engulf cytosolic material. LC3-lipidation, the signature of autophagosome formation, results from a complex ubiquitin-conjugating cascade orchestrated by the ATG16L1 protein, whose regulation is unknown. Here, we identify the Gigaxonin-E3 ligase as the first regulator of ATG16L1 turn-over and autophagosome production. Gigaxonin interacts with the WD40 domain of ATG16L1 to drive its ubiquitination and subsequent degradation. Gigaxonin depletion induces the formation of ATG16L1 aggregates and impairs LC3 lipidation, hence altering lysosomal fusion and degradation of the main autophagy receptor p62. Altogether, we demonstrate that the Gigaxonin-E3 ligase controls the production of autophagosomes by a reversible, ubiquitin-dependent process selective for ATG16L1. Our findings unveil the fundamental mechanisms of the control of autophagosome formation, and provide a molecular switch to fine-tune the activation of autophagy.

## Abstract (web summary)

Membrane elongation is fundamental to autophagy and is controlled by an ubiquitin-conjugating cascade orchestrated by ATG16L1. Here, the authors identify that the E3 ligase Gigaxonin regulates autophagosome formation by controlling ATG16L1 turnover.

## Introduction

Autophagy is an essential degradative pathway that delivers cytoplasmic components to lysosomes for degradation. Evolutionarily conserved, this complex machinery is activated to recycle a wide range of substrates in normal conditions and to promote the degradation of damaged components (dysfunctional organelles, protein aggregates) in diseases^1^. Therefore, alteration of autophagy perturbs cellular homoeostasis and important physiological processes^2^, and it is associated with various pathological conditions, including cancer and neurodegenerative diseases^3–5^. Macroautophagy (hereafter referred to as autophagy) is characterised by the nucleation of a double-membrane fragment (phagophore) around the material to be degraded, which elongates to form a complete autophagosome and subsequently fuses to a lysosome^6,7^.

The mechanisms driving membrane expansion are key in autophagy. The molecular determinants of membrane elongation are complex and involve two highly conserved ubiquitin-like (UBL) conjugation systems, ATG12 and LC3 (the mammalian homologue of the yeast Atg8)^8,9^. Structurally related to ubiquitin, ATG12 and LC3 are transferred by E1- and E2-like enzymes to their final substrates. The covalent conjugation of ATG12 to ATG5 generates the E3 ligase activity necessary for the last step of ATG8/LC3 conjugation to phosphatidylethanolamine (PtdEth) on the nascent membranes^10^.

Orchestrating this cascade at the site of the nascent phagophore, ATG16L1^11,12^ is a key determinant of autophagy elongation. Indeed, ATG16L1 interacts with the conjugate ATG12-ATG5 to form a multimeric structure^13^ and triggers the binding of the complex to the membrane. Through the subsequent interaction of ATG12 with LC3-conjugated-ATG3^14,15^, ATG16L1 specifies the site of LC3 lipidation onto nascent membranes^16^. Several studies in yeast and mammalian cells have shown that alterations in ATG16L1, either using genetic mutants or the overexpressed protein, all result in impaired localisation of ATG12-ATG5 to the phagophore and failure in ATG8/LC3 lipidation onto the membranes, leading to inhibition of autophagosome formation^13,17–20^. Likewise, forced localisation of ATG16L1 to the plasma membrane has been shown to be sufficient to promote ectopic LC3 lipidation at the cell surface^17^. The biological importance of ATG16L1 was further evidenced in vivo, where *Atg16L1*^*-/-*^ mice, defective in autophagosome formation, did not survive neonatal starvation and died within 1 day of delivery^19^. Thus, regulation of the scaffold ATG16L1 protein constitutes not only a fundamental question to apprehend the complex dynamics of autophagic activity but also represents a substantial target for therapy to activate autophagy in disease. Post-translational modifications (PTMs) of ATG proteins are essential in modulating their activity. While more than 300 PTMs of autophagic proteins have been characterised^21,22^, very little is known about ATG16L1, and only Ser2878 phosphorylation has been evidenced in acute intestinal inflammation^23^.

Here we identify Gigaxonin^24^, an E3 ligase mutated in a fatal neurodegenerative disease called giant axonal neuropathy (GAN)^25^, as the first regulator of ATG16L1. Gigaxonin poly-ubiquitinates and controls the degradation of ATG16L1, and is essential to activate autophagy. Accumulation of ATG16L1, as a result of Gigaxonin depletion, alters early events prior to the docking of the autophagy elongation conjugate to the phagophore, and diminishes fusion to the lysosome and degradation of the autophagy receptor p62. We demonstrate that Gigaxonin depletion inhibits autophagosome synthesis, which is rescued upon reintroduction of the E3 ligase. Altogether, our data unveil the regulatory mechanism that drives the dynamics of autophagosome formation by ATG16L1, and position Gigaxonin as a significant therapeutic target to modulate autophagy activity in disease.

## Results

### Gigaxonin interacts with the WD40 domain of ATG16L1

**Fig. 1:**
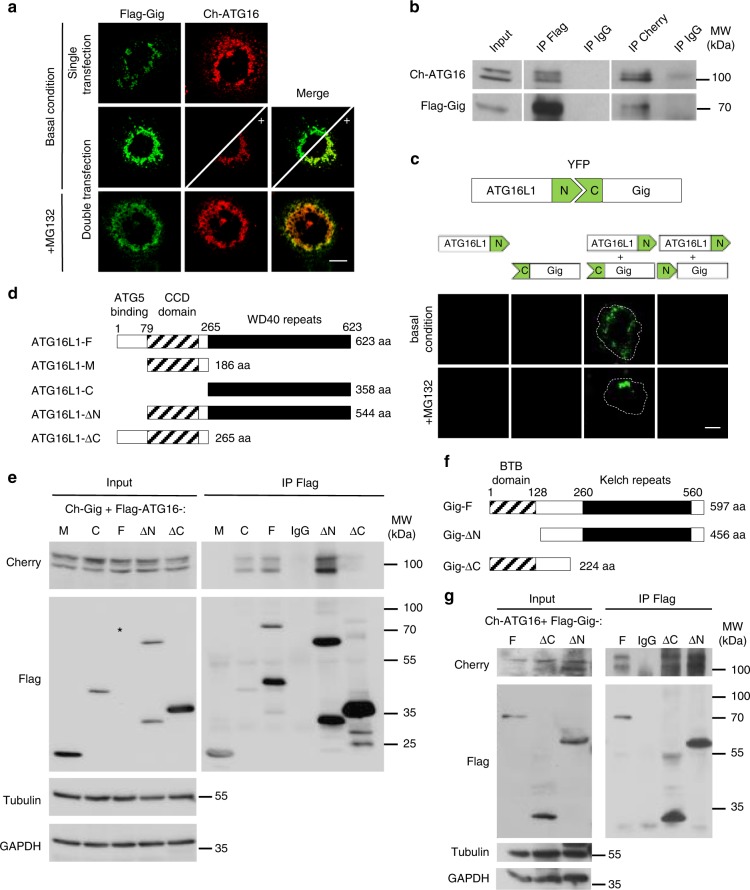
Gigaxonin interacts with the WD40 domain of ATG16L1. **a** Immunofluorescence of COS-7 cells expressing Cherry-ATG16L1 (Ch-ATG16) with or without Flag-Gigaxonin (Flag-Gig). Boosting of Ch-ATG16 intensity (as shown by plus sign in the bottom panel delineated by the diagonal line) in normal condition, and MG132 treatment condition revealed a colocalisation between Gigaxonin and ATG16L1. **b** Reverse immunoprecipitation experiment was performed in COS-7 cells transfected with Ch-ATG16 and Flag-Gig and treated with MG132. Gigaxonin and ATG16L1 complexes were recovered using anti-flag (IP Flag) and anti-cherry (IP Cherry) antibodies and revealed by immunoblotting with Cherry/Flag antibodies. Immunoprecipitation with anti-IgG antibodies (IP IgG) served as negative control. **c** Interaction between ATG16L1 and Gigaxonin was demonstrated by bimolecular fluorescence complementation (BiFC) assay, in transfected COS-7 cells ± MG132. Interaction is visualised by the physical interaction between the N-terminal portion of YFP carrying construct (ATG16L1-N) and the C-terminal YFP construct (C-Gig). Negative controls include single transfection of ATG16L1-N, C-Gig or cotransfection of ATG16L1-N with N-Gig constructs. The dashed lines delineate the cells. **d** Schematic representation of the mouse ATG16L1 (ATG16L1-F) and its deletion constructs. N-terminal, central coiled-coil and C-terminal WD40 repeat domains are indicated in white, grey stripes and black, respectively. **e** The domain of interaction of ATG16L1 with Gigaxonin was evidenced by immunoprecipitation. Full-length and deletion constructs of Flag-ATG16 were transfected in COS-7 cells with Ch-Gig in presence of MG132. Please not that the Full-length ATG16L1 band (black star in the input panel) is enriched in pull down condition (in the IP Flag panel). Immunoprecipitation using anti-Flag antibodies evidenced Gigaxonin in the immunocomplexes formed by the Full-length-, C- and ∆N- ATG16L1 proteins. Negative controls include immunoprecipitation of the Ch-Gig + Flag-ATG16L1-F cell lysate with anti-IgG antibodies (IP IgG). **f** Schematic representation of the human Gigaxonin (Gig-F) with its N-terminal BTB (stripes) and C-terminal Kelch (black) domains and the BTB and Kelch deletion constructs. **g** Immunoprecipitation of Flag-Gig complexes identified Ch-ATG16 with both deletion constructs of Gigaxonin, Scale bar: 10 µm

Gigaxonin was proposed as a possible partner of ATG16L1, in a study reconstructing the autophagy interaction network^26^. To determine whether this interaction occurs with biological significance, we combined cellular assays for constructs bearing the Cherry-ATG16L1 (Ch-ATG16) and Flag-tagged Gigaxonin (Flag-Gig). Strikingly, immunofluorescence of COS cells expressing both constructs (Fig. 1a) revealed that ATG16L1 was degraded upon Gigaxonin expression. Restoring ATG16L1 content using the proteasome inhibitor MG132, or focusing on the residual ATG16L1, evidenced a colocalisation between Gigaxonin and ATG16L1. We expanded on this to demonstrate the physical interaction between ATG16L1 and Gigaxonin in COS cells, by reverse immunoprecipitation experiments, in which we stabilised ATG16L1 with proteasome inhibitor (Fig. 1b). To further confirm their direct interaction, we performed a bimolecular fluorescence complementation (BiFC) assay, which relies on the reconstitution of a fluorescent reporter protein in live cells, as the result of the physical proximity of its complementary fragments upon interaction of the proteins fused to the fragments. This live assay revealed a specific interaction between Gigaxonin and ATG16L1, which was promoted by proteasome inhibition (Fig. 1c). ATG16L1 is composed of three main structural domains: an N-terminal binding fragment followed by a central self-oligomerization coiled-coil domain (CCD) and a C-terminal WD40 domain (Fig. 1d). To determine which domain of ATG16L1 interacts with Gigaxonin, we performed co-immunoprecipitation with Flag-tagged ATG16L1 deletion constructs (Fig. 1e). Cherry-Gigaxonin (Ch-Gig) was identified in immunocomplexes with the full-length ATG16L1 (F), the ATG16L1-C (C) and the ATG16L1-ΔN (ΔN) proteins, and not the other deletion products, demonstrating a specific interaction of Gigaxonin with the C-terminal WD40 domain of ATG16L1. Similarly, we pulled down Ch-ATG16L1 with truncated portions of Gigaxonin to reveal that both BTB and Kelch domains, or alternatively a common domain lying in the linker domain can interact with ATG16L1 (Fig. 1f, g).

### Gigaxonin mediates ubiquitin-dependent degradation of ATG16L1

**Fig. 2:**
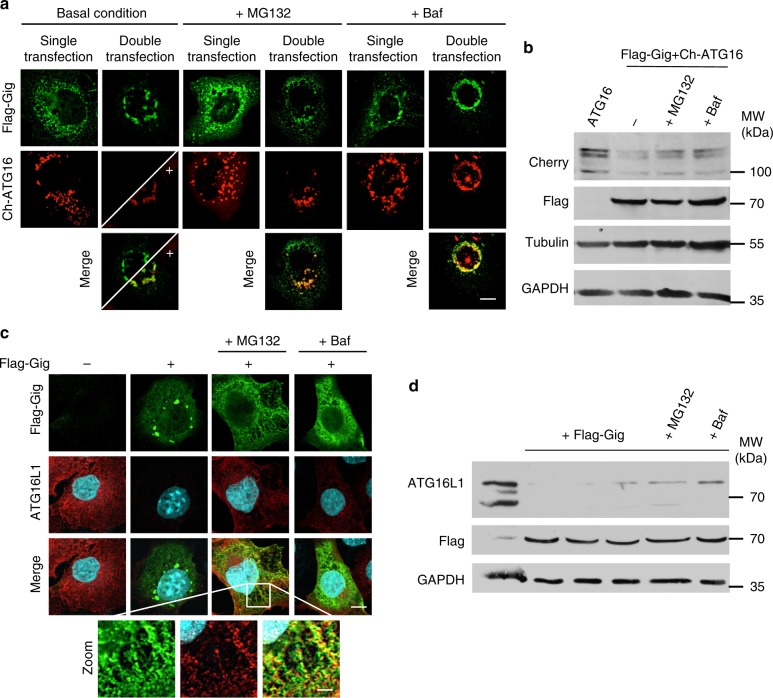
Gigaxonin induces the degradation of ATG16L1, through the proteasome and the lysosomal pathways. **a** Immunofluorescence of COS-7 cells transfected with Flag-Gigaxonin and Cherry-ATG16L1 alone (single transfection) or together (double transfection), in basal condition, or treated with MG132 or Bafilomycin A1 (Baf). Gigaxonin-dependent degradation of ATG16L1 is rescued upon both treatments. **b** Immunoblotting of cell extracts prepared as in **a** to show the proteasome and autophagy-dependent degradation of ATG16L1 by Gigaxonin. Immunofluorescence (**c**) and immunoblotting (**d**) showed that endogenous ATG16L1 protein is greatly diminished in Gigaxonin transfected cells, and partially restored under MG132 or Bafilomycin A1 treatment. Scale bar: 10 µm and 50 µm for zoom pictures

Considering the physical interaction between Gigaxonin and ATG16L1 and the substantial effect in ATG16L1 clearance (Fig. 1), we further examined the role of the Gigaxonin-E3 ligase in ATG16L1 turn-over. To overcome possible bias due to transfection, we studied Gigaxonin function on overexpressed ATG16L1 (Cherry-ATG16L1) (Fig. 2a, b) and on the endogenous ATG16L1 (Fig. 2c, d). We demonstrated that while Cherry-ATG16L1 was completely degraded by ectopic Gigaxonin, the inhibition of the proteasome (by MG132) or lysosomal proteolysis (using Bafilomycin A1) restored to some extend Cherry-ATG16L1 levels, as revealed by immunofluorescence and immunoblotting (Fig. 2a, b). Importantly, we confirmed the role of Gigaxonin in mediating the degradation of the endogenous ATG16L1, through the proteasome and autophagy routes (Fig. 2c, d).

**Fig. 3:**
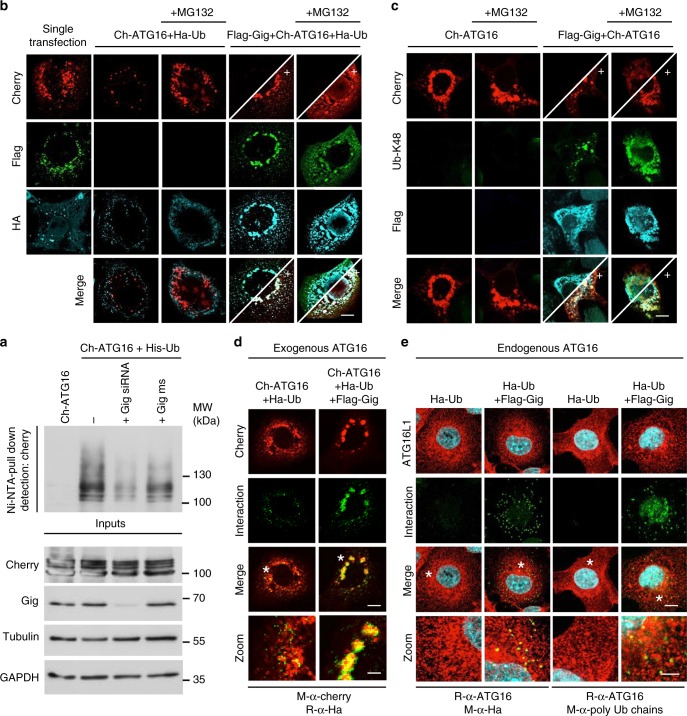
Gigaxonin promotes the ubiquitination of ATG16L1. **a** In vivo ubiquitination assay for ATG16L1. COS-7 cells were transfected with Ch-ATG16 alone, or in combination with His-Ubiquitin construct (His-Ub) in the presence or absence of short interfering RNA (siRNA) against endogenous Gigaxonin (Gig siRNA) or the mismatch counterpart (Gig ms) and treated with MG132. The pull down of ubiquitinated ATG16L1 is performed in denaturing conditions using nickel agarose beads. **b** Colocalisation of ubiquitin with ATG16L1 upon Gigaxonin expression. COS-7 cells were transfected with Flag-Gig, Ch-ATG16 and HA-Ubiquitin (Ha-Ub) constructs ± MG132. The expression of Gigaxonin induces a drastic degradation of Ch-ATG16, and all remaining ATG16L1 (as revealed by +) colocalises with Gigaxonin and ubiquitin. **c** Gigaxonin promotes the K48-ubiquitination of ATG16L1. Specific labelling of K48 ubiquitin chain did not show substantial colocalisation of this ubiquitin motif on ch-ATG16, when transfected alone in COS-7 cells. In presence of Flag-Gig, Ch-ATG16L1 was degraded and fully decorated by K48-ubiquitin, as shown by the colocalisation of anti-K48 antibodies with ch-ATG16 in boosted pictures ± MG132. **d** Additional evidence for the ubiquitination of ATG16L1 by the Gigaxonin-E3 ligase using proximity ligation assay (PLA). The physical proximity between ubiquitin and ATG16L1 was revealed after transfection of HA-Ubiquitin with Cherry-ATG16L1 ± Flag-Gigaxonin under MG132 treatment. The specificity of the reactions of the PLA assay is provided in Supplementary Fig. 1a. **e** The poly-ubiquitination of the endogenous ATG16L1 is promoted by Gigaxonin. PLA was performed on COS-7 cells, transfected only with the HA-Ub ± Flag-Gigaxonin under MG132 treatment. The specificity of the reactions of the assay is provided in Supplementary Fig. 1b. Left panel: PLA amplification using anti-HA/anti-ATG16L1 antibodies. Right panel: The PLA amplification using anti-polyubiquitin chains/anti-ATG16L1 antibodies. Scale bar: 10 µm and 30 µm for zoom pictures

Considering the role of Gigaxonin as a substrate adaptor of Cul3-E3 ubiquitin ligases^27^, we next addressed whether the degradation of ATG16L1 by Gigaxonin is mediated by its ubiquitination activity. Toward this aim, we conducted an in vivo ubiquitination assay for ATG16L1, and determined the effect of Gigaxonin, by depleting the endogenous Gigaxonin protein. Considering the ability of the WD40 domain of ATG16L1 to transiently bind ubiquitin^28^, we performed the ubiquitin pull down experiments under denaturing conditions. Thus, the covalent ubiquitination of the Cherry-ATG16L1 protein was assessed after transfection with a His-Ubiquitin construct in presence or absence of Gigaxonin short interfering RNAs (siRNAs), and the subsequent pull down of ubiquitinated proteins with nickel agarose beads (Fig. 3a). This experiment demonstrates that while Gigaxonin is not the only E3 ligase to promote ATG16L1 covalent ubiquitination in COS cells, its repression diminishes this PTM, hence providing the direct evidence of the role of Gigaxonin in promoting ATG16L1 ubiquitination. To further confirm this result, we combined colocalisation experiments (Fig. 3b, c) and Proximity Ligation Assay (PLA) (Fig. 3d, e). First, we employed a cell-based analysis for in vivo ubiquitination by co-transfecting HA-Ubiquitin (Ha-Ub) with Ch-ATG16 ± Flag-Gig (Fig. 3b). This analysis revealed that while a small proportion of ATG16L1 was ubiquitin positive when expressed alone, all residual ATG16L1 was co-labelled with ubiquitin upon Gigaxonin expression, hence confirming the Gigaxonin-dependent ubiquitination of ATG16L1 (Fig. 3b). Moreover, staining with anti-K48-ubiquitin antibodies revealed a complete colocalisation of this Ub chain with the residual ATG16L1 upon Gigaxonin expression, thereby suggesting that Gigaxonin induces the specific elongation of K48-type ubiquitin chain onto ectopic ATG16L1 (Fig. 3c). Furthermore, we performed a proximity ligation assay (PLA) for ubiquitin and ATG16L1 (Fig. 3d, e). Combining a wide range of negative and positive controls, we further confirmed the interaction between ATG16L1 (exogenous and endogenous) and Gigaxonin, and demonstrated the specificity of the PLA assay (Supplementary Fig. 1a, b). This analysis demonstrated that while a minor pool of ectopic ATG16L1 is decorated with ubiquitin, Gigaxonin dramatically increases this colocalisation (Fig. 3d). Indeed, we showed that Gigaxonin enhanced the fluorescence intensity, as a result of an increased proximity between HA tagged ubiquitin and Cherry-ATG16L1. Furthermore, the comparison between the PLA and Ch-ATG16L1 pattern revealed that while only a sub-population of ectopic ATG16L1 is co-labelled with ubiquitin, the presence of Gigaxonin decorated all ATG16L1 with ubiquitin, hence supporting the dramatic effect of Gigaxonin on ATG16L1 clearance. Importantly, we also confirmed that Gigaxonin greatly increases the proximity between ubiquitin/polyubiquitin chains and the endogenous ATG16L1, hence supporting an activity of Gigaxonin in the poly-ubiquitination of endogenous ATG16L1 (Fig. 3e).

Altogether, our data combine to evidence that the turn-over of ATG16L1 is controlled by Gigaxonin, through its K48-poly-ubiquitination and degradation by the proteasome and autophagy.

### Gigaxonin depletion induces ATG16L1 aggregation in neurons

**Fig. 4:**
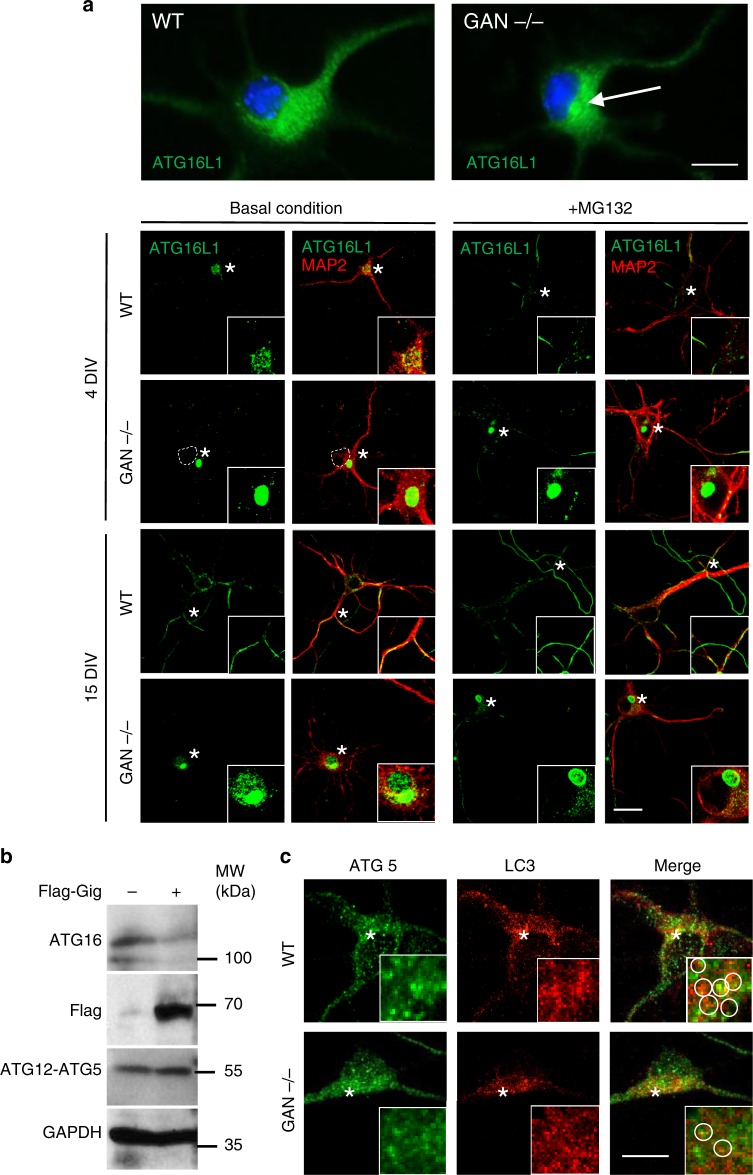
ATG16L1 accumulates in *GAN*^-/-^ cortical neurons and impairs the autophagy elongation conjugate. **a** MAP2 positive neurons, prepared from cortices of E15.5 GAN embryos were immunostained with ATG16L1 antibody after 4 and 15 *div* (days in vitro) ± proteasome inhibitor. Top panel shows an enlarge picture of the single perinuclear bundle of ATG16L1 found in the soma of *GAN*^-/-^ cells. Dashed line shows the nucleus. Scale bar: 10 µm for upper panel and 20 µm for bottom panel. **b** The effect of Gigaxonin in the conjugation of ATG12-ATG5 was evidenced in COS-7 cells. Transfected Flag-Gigaxonin degraded endogenous ATG16L1 but did not alter the formation of ATG12-ATG5 elongation complex, which migrates at 55 kDa. **c** ATG5 does not aggregate in Gigaxonin-null neurons. The visualisation of the docking of endogenous ATG5 to autophagosomes was hampered by the high density of LC3 dots in cortical neurons, but would reveal a decreased density around autophagic structures in the *GAN*^-/-^ neurons (inserts: circles drawn to highlight LC3-ATG5 proximity in individual dots). Scale bar: 10 µm

In light of the important role of Gigaxonin in controlling ATG16L1 degradation, we investigated the effect and the significance of this regulation in a physiological context. For that purpose, we developed a cellular model deficient for Gigaxonin, from the knock-out mouse depleted in the *GAN* gene^29^, which causes a fatal and recessive neurodegenerative disorder called giant axonal neuropathy in human^24^. In agreement with our results, we confirmed that ATG16L1 is controlled by Gigaxonin in primary neuronal cells. Examination of MAP2 positive control cells revealed a spatial distribution/biogenesis of the endogenous ATG16L1 over time, which was mostly located in the soma at 4 *div* (days in vitro) and within neurites at 15 *div* (Fig. 4a). On the contrary, *GAN*^*-/-*^ neurons exhibited large perinuclear bundles of ATG16L1 within the soma at 4 *div*, with a pronounced increase in abundance. At 15 *div*, the ATG16L1 aggregates persisted but were not distributed in neurites, as in wild type cells. To assess for the contribution of the proteasome in the impaired degradation of ATG16L1 upon Gigaxonin depletion, we compared ATG16L1 distribution and abundance in the presence of MG132. Interestingly, in treated controls cells, ATG16L1 was mostly found in neurites at 4 *div*, similarly to older neurons, which suggests a constitutive and active degradation of ATG16L1 locally within neurites in early developmental stages. Strikingly, the *GAN*^*-/-*^ treated neurons were able to respond to some extent to the proteasome inhibition in neurites, but did not exhibit overt exacerbation of ATG16L1 bundling in the soma, at any time point. Collectively, our data suggest that Gigaxonin may constitute the main or possibly the unique E3 ligase for ATG16L1 in the soma. Moreover, proteasome inhibition was not effective in generating ATG16L1 aggregates in the soma of wild type neurons, which emphasises the potent role of Gigaxonin in degrading ATG16L1 through several routes as demonstrated in this study.

### Gigaxonin alters the elongation complex but not ATG12-ATG5

ATG16L1 is essential in the elongation phase of autophagy. Indeed, it forms a ternary complex with the preformed ATG12-ATG5 conjugate^11^, which constitutes the E3 ligase promoting the LC3 conjugation to the nascent autophagic membrane^8^. Crucial for the recruitment of ATG12-ATG5 to the phagophore^17^, ATG16L1 determines the site of LC3 lipidation and therefore primes the elongation of the autophagosome. Considering our results on the ubiquitin-dependent degradation of ATG16L1 by Gigaxonin (Figs. 1–3) and its aggregation in the absence of Gigaxonin (Fig. 4a), we addressed whether the ATG12-ATG5 elongation conjugate was altered in Gigaxonin-depleted neurons. Firstly, we revealed that Gigaxonin overexpression did not alter the formation of the ATG12-ATG5 conjugates in COS cells (Fig. 4b), nor induce their aggregation (Fig. 4c) in *GAN*^*-/-*^ neurons. These findings further support the specificity of Gigaxonin action on ATG16L1 and recapitulate previous studies showing that neither ATG16L1 overexpression nor repression alters ATG12-ATG5 conjugation^17,19^. Extremely challenging to evaluate with endogenous proteins in primary neurons, we could not clearly evidence that accumulation of ATG16L1 precludes the docking of the elongation conjugate to the membranes, as shown with the decrease of GFP-ATG5 dots upon ATG16L1 modulation^17,19^. Still, we noticed that LC3 dots were less frequently decorated by endogenous ATG5 in *GAN*^*-/-*^ neurons, in comparison to control cells (enlarged pictures in Fig. 4c). Our data pinpoints a unique role of Gigaxonin in controlling the autophagy elongation complex, by degrading ATG16L1.

### GAN neurons show defects in autophagosome synthesis

**Fig. 5:**
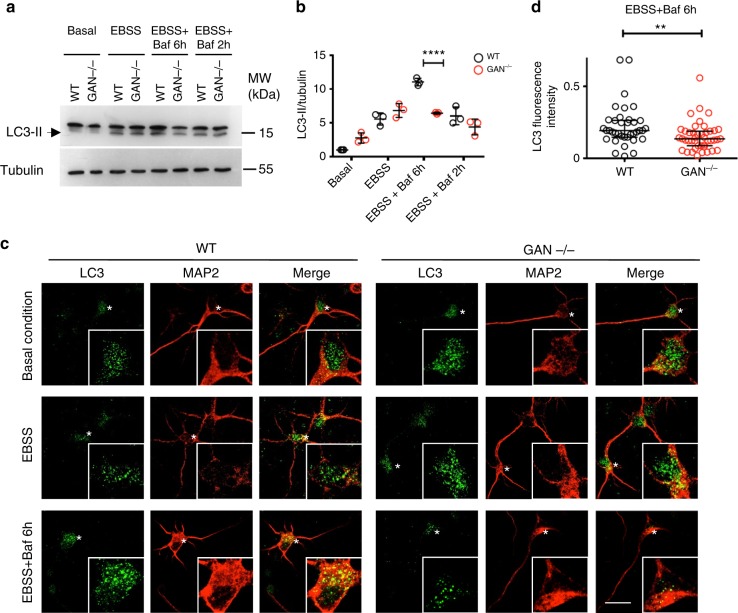
Gigaxonin depletion causes a defect in autophagosome production. **a–d** Wild-type and *GAN*^-/-^ cortical neurons were cultured for 4 *div* under basal condition, or with different treatments to evaluate autophagy activity. Starvation was induced with EBSS, autophagosome-lysosome fusion was blocked with Bafilomycin A1 (Baf). **a** Immunoblotting of cortical neurons to show the lipidation of LC3 under different conditions, in control and mutant neurons. **b** Quantification of the lipidation of LC3 was measured with the LC3-II to tubulin ratio, and expressed as a fold increase over the control level in the basal condition. The defect in autophagy in *GAN*^*-/-*^ neurons, evidenced by the decreased LC3 lipidation in EBBS + Baf 6h condition was further defined as a decreased production of autophagosomes, by comparing the EBBS + Baf ratio at two different times (2 h and 6 h of treatment). WT and mutant values are represented in black and red circles, respectively. *n* = 3 independent experiments and values are means ± SD. Differences between WT and mutant values are only significant for the (EBSS + Baf 6h) condition ****P < 0.0001 using two-way ANOVA test (Bonferroni post-hoc test). Other multiple comparisons, not presented on the figure for clarity purpose are: (1) control values: ****P < 0.0001 between basal and EBSS, ****P < 0.0001 between EBSS and (EBSS + Baf 6h); ****P < 0.0001 between (EBSS + Baf 6h) and (EBSS + Baf 2h); (2) for the mutant cells: ***P < 0.001 between basal and EBSS. **c** The decrease of autophagosomes, visualised by the reduced density of LC3 dots, was evidenced in *GAN*^*-/-*^ neurons labelled with the MAP2 antibodies under EBBS + Baf 6h treatment. Scale bar: 25 µm. **d** The quantification of LC3 dots was obtained by measuring the fluorescence intensity of LC3 staining in EBSS + Baf 6h condition (*n* = 37 for WT cells represented in black circles, *n* = 43 cells for mutant cells represented in red circles, from three independent experiments). Individual measures and medians with interquartile range are represented; statistical significance was obtained using the two-tailed Mann–Whitney test with a **P < 0.01 value

To directly demonstrate the role of Gigaxonin in the early steps of autophagosome maturation, we investigated the different phases of the autophagy pathway in *GAN*^*-/-*^ neurons. For that purpose, we compared the responsiveness of control and mutant cells to an inducer of autophagy (EBSS) and to a drug blocking the autophagosome-lysosome fusion (Bafilomycin A1). First, the quantification of LC3 lipidation (Fig. 5a, b) and intensity (Fig. 5c, d) was measured to ascertain the number of autophagosomes. This analysis revealed that, as control cells, *GAN*^*-/-*^ neurons were able to produce autophagic vesicules in basal condition and upon serum deprivation (Basal and EBBS conditions in Fig. 5a–c). Nevertheless, conditions in which autophagy induction was combined to a blocking of autophagosome-lysosome fusion evidenced a severe alteration of autophagosome synthesis in absence of Gigaxonin. Indeed, control cells were able to further increase autophagosome number when Bafilomycin A1 was applied for 6 h, while *GAN*^*-/-*^ neurons did not (EBSS + Baf 6h in Fig. 5a–d). The decrease in the net production of autophagosome synthesis was further confirmed by the inability of Gigaxonin-null neurons to promote LC3 lipidation between 2 h and 6 h in EBBS and Bafilomycin A condition (EBSS + Baf 6h and EBSS + Baf 2h in Fig. 5a, b). We complemented this analysis by examining p62, which is the main selective autophagy receptor and is conventionally used as a marker of effective autophagic degradation. In control cells, p62 accumulated only in Bafilomycin A1 treatment, as a result of the impairment of the degradation of autophagosome content by lysosomal enzymes (as seen in Baf and EBSS + Baf 6h conditions in Supplementary Fig. 2). On the contrary, p62 was shown to accumulate in all conditions in *GAN*^*-/-*^ neurons, hence demonstrating a defect in basal autophagy when Gigaxonin is absent. Considering our findings of an impairment of autophagosome synthesis in *GAN*^*-/-*^ neurons (Fig. 5), p62 most probably decorates phagophores, which failed to elongate and therefore provoked p62 accumulation. To verify this hypothesis, we examined the late stage of autophagosome maturation, and showed that, contrary to control neurons, neither LC3 positive membranes nor p62 aggregates colocalized efficiently with lysosomes in basal condition (Supplementary Fig. 3). In addition, in conditions which favour the fusion as a result of autophagy induction in control cells, *GAN*^*-/-*^ neurons were still unable to fuse their p62 structures to lysosomes (EBSS in Supplementary Fig. 4), hence demonstrating a severe blocking of the maturation of autophagic structures upon Gigaxonin depletion.

Collectively, our data evidence that Gigaxonin depletion alters early steps of autophagy, by impairing autophagosome formation.

### Gigaxonin overexpression restores autophagy in GAN neurons

**Fig. 6:**
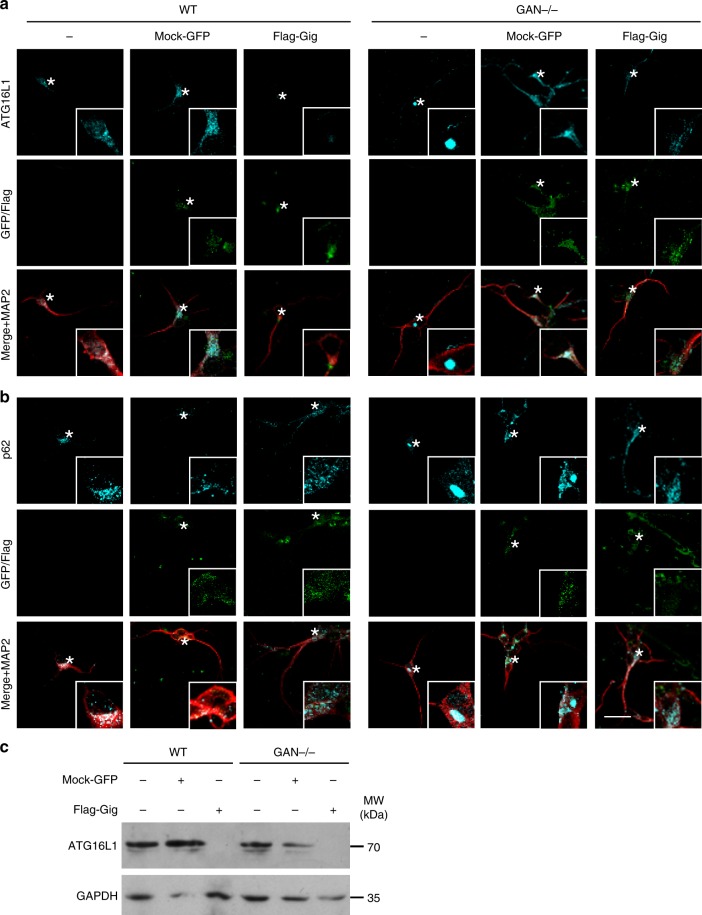
Early and late autophagy steps are rescued by lentiviral-Gigaxonin in *GAN*^-/-^ neurons. Immunostaining (**a**) and immunoblots (**c**) for ATG16L1 were performed on 4 *div* control and mutant neurons, after transduction with mock-GFP or Flag-Gigaxonin lentivirus. Both normal and bundled ATG16L1 were cleared upon expression of Gigaxonin in control and *GAN*^-/-^ neurons, respectively. **b** Gigaxonin expression restored p62 levels in mutant cells, supporting an effective maturation of autophagosomes and autophagy degradation. Scale bar: 20 µm

To confirm that Gigaxonin controls autophagosome production through the regulation of ATG16L1 turn-over in a physiological context, we performed a rescue experiment in *GAN*^*-/-*^ neurons. Using a lentiviral approach, we demonstrated that Gigaxonin overexpression, but not the mock counterpart, was able to reduce endogenous levels of ATG16L1 in control cells, further corroborating the control of its abundance by Gigaxonin in primary neurons (Fig. 6a, b). Robustly, Gigaxonin totally cleared ATG16L1 bundles in *GAN*^*-/-*^ neurons. We next suggested that this rescue on ATG16L1 could convey into a restoration of autophagy flux in *GAN*^*-/-*^ neurons, by demonstrating a restoration of p62 degradation (Fig. 6a). Thus, our data confirm the action of the Gigaxonin-E3 ligase in ATG16L1 turn-over in primary neurons, but also support the notion of a restoration of autophagosome maturation upon Gigaxonin expression.

**Fig. 7:**
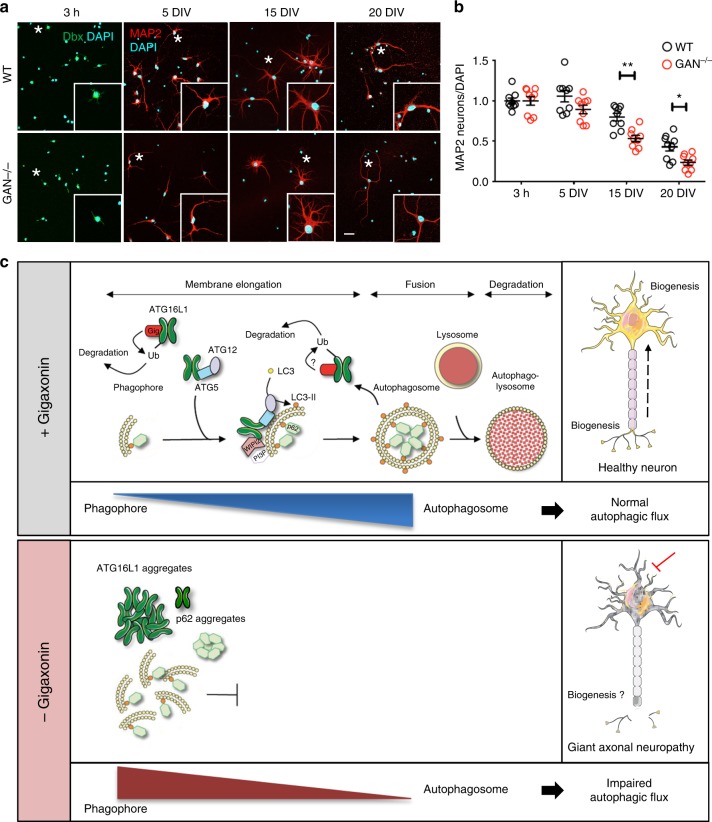
Progressive degeneration of *GAN*^-/-^ neurons and model of action of Gigaxonin. **a** Immunostaining with Doublecortine (Dbx) and MAP2 identified neurons from control and *GAN*^-/-^ mice after 3 hours, 5, 15 and 20 *div*. Scale bar: 25 µm. **b** Neurodegeneration of *GAN*^-/-^ neurons was evidenced from 15 *div* onwards, as measured by the decreased proportion of neurons (as expressed by a MAP2 to DAPI ratio), relatively to the 3 h time point (Dbx to Dapi ratio). WT and mutant cells are represented in black and red circles, respectively. *n* = 3 independent experiments with triplicate measures (161–821 DAPI-positive cells counted per measure). Individual measures and means ± SEM are represented; **P < 0.01 and *P < 0.05 values at 15 and 20 *div*, respectively using the two-way ANOVA test (Bonferroni post-hoc test). **c** Schematic model of Gigaxonin action. Top panel: Gigaxonin controls the steady-state level of ATG16L1, by interacting with its C-terminal WD40 domain and promoting its K48 poly-ubiquitination and clearance by the proteasome and the autophagy pathways. ATG16L1 binds to the ATG12-ATG5 elongation conjugate and targets it to the nascent autophagic membrane, through its interaction with WIPI2. Thus, the E3 ligase activity of the ATG12-ATG5-ATG16 complex lipidates LC3 onto the membranes, allowing the elongation of the phagophore. The mature autophagosome sequesters cytosolic materials, including p62 bound cargo, which are degraded upon fusion to the lysosome. Thus, by controlling ATG16L1 steady-state, the Gigaxonin-E3 ligase promotes autophagosome production and ensures a normal autophagic flux within cells. In a physiological context, autophagosome biogenesis occurs at the neurite tip but also in the soma of primary neurons. Bottom panel: Gigaxonin depletion in primary neurons induces aggregation of ATG16L1, without impairing the formation of the ATG12-ATG5 conjugate. We propose that the impairment in LC3 lipidation observed in *GAN*^-/-^ neurons is due to a defect in anchoring ATG12-ATG5 to the autophagic membranes. Overall, Gigaxonin depletion alters autophagosome synthesis and causes an abnormal accumulation of the main autophagy receptor p62, hence impairing the autophagic flux. ATG16L1 and P62 accumulation are localised within the soma, opening interesting perspectives on the study of autophagy compartimentalisation within neurons, and on its role in human neuropathy, as exemplified in GAN. (Image templates coming from Servier Medical Art (https://na01.safelinks.protection.outlook.com/?url=http%3A%2F%2Fsmart.servier.com%2F&data=02%7C01%7Caurora.scrivo%40einstein.yu.edu%7Cc44f29f2622d461b35a508d654507f0e%7C04c70eb48f2648079934e02e89266ad0%7C1%7C0%7C636789102036800397&sdata=zpNk9F6HX1iwcI%2Fdm5MXXUj3yRUvykH64lwEWuH1J8U%3D&reserved=0)

Considering that loss of Gigaxonin causes a neurodegenerative disorder^24^, our results suggest that autophagosome membrane elongation is likely to be impaired in patients. While this cannot be assessed in humans, we have generated a neuronal model for GAN to mimick Gigaxonin loss-of-function as a very valuable biological system in determining the contribution of autophagy in the disease. Interestingly, we revealed a progressive neurodegeneration in *GAN*^*-/-*^ cortical neurons at late stage (15 *div*, Fig. 7a, b), subsequently to the apparition of autophagy deficits (5 *div*). Thus, targeting ATG16L1 may represent an exciting therapeutic avenue for GAN, but also for other conditions with alterations in the initial steps of autophagy activation, for which targeted approaches are needed to reduce deleterious side effects.

## Discussion

The ATG16L1 protein orchestrates the two UBL systems required for membrane elongation during autophagosome formation, but its control and dynamics are unknown. In this study, we identify the Gigaxonin-E3 ligase as the first regulator of ATG16L1 turn-over, essential in ensuring ATG16L1 functions in phagophore expansion (Fig. 7c). First, Gigaxonin interacts with the C-terminal WD40 domain of ATG16L1 to promote its ubiquitination and degradation by the proteasome and the autophagy pathways. Second, Gigaxonin depletion induces a massive aggregation of ATG16L1 in primary neurons and inhibits autophagy flux by impairing phagophore elongation. We propose a pivotal role of the Gigaxonin-E3 ligase in controlling autophagosome formation, hence ensuring the fine-tuning of the activation of autophagy (Fig. 7c).

Up to now, no E3 ligase was shown to act on membrane elongation^30,31^ and no ubiquitin PTM has been identified for ATG16L1^21,22^. Only one polymorphic variant of ATG16L1, associated to the inflammatory Crohn’s disease, has shown an increased sensitivity to caspase-3-mediated processing^32^, which can be modulated by phosphorylation during inflammation^23^. The regulation of ATG16L1 by Gigaxonin is highly robust, as revealed by the complete clearance of ATG16L1 in cell lines, and the absence of increased ATG16L1 bundling upon proteasome inhibition in *GAN*^*-/-*^ neuronal cells.

In this study, we demonstrate an impairment of autophagosome production in absence of Gigaxonin, which causes defective autophagic degradation with decreased fusion to lysosomes and p62 accumulation. The specificity and efficacy of Gigaxonin was further corroborated in a rescue experiment showing not only a clearance of ATG16L1 upon Gigaxonin overexpression in control and mutant neurons, but also a restoration of p62 degradation in GAN cells. Strikingly, our phenotypes are similar to the effects caused by the alterations of ATG16L1 and WIPI2 (a protein which allows ATG16L1 localisation to the phosphatidylinositol 3-phosphate, Ptdlns3P, generated on membranes upon autophagy initiation)^33^. Indeed, autophagosome formation is impaired upon WIPI2 depletion^34,35^ and ATG16L1 overexpression^17,35^, but also ATG16L1 depletion^19^. It is likely that the level of lipidated LC3 that we observe in basal condition in *GAN*^*-/-*^ cells reflects an accumulation of incomplete autophagosomes. Indeed, a similar LC3-II increase was reported by other groups^34,35^ and was associated with the presence of phagophores and open autophagosomes in *Atg16L1* ^-/-^ MEF cells on electron micrographs^34^. Thus, we have revealed the mechanism of regulation on ATG16L1 that controls the early steps of phagophore elongation. In absence of Gigaxonin, ATG16L1 accumulates and would, as previously reported, exert a dominant effect on the localisation of ATG12-ATG5 to the membranes, without affecting the formation of the conjugate (ref. ^17^ and our data).

We were intrigued by the localisation of the ATG16L1 bundles within GAN^-/-^ neurons. Indeed, ATG16L1 bundles are mostly present in a perinuclear position in the soma, and while not totally absent, rarely seen within neurites. Autophagosome biogenesis has been shown to be spatially regulated along the axons; it occurs distally in the neurite tip and the autophagosomes mature and fuse to the lysosome while undergoing a retrograde transport towards the soma^36–39^. Still, some autophagosome formation was observed in the soma^37^, showing specificity in their maturation, localisation and dynamics^40^ (Fig. 7c). While several functions of axonal autophagy have been identified, including axonal homoeostasis and presynaptic functions, nothing is known about its role in the soma^41,42^. Thus, Gigaxonin will contribute to deciphering the meaning of the high degree of compartimentalisation of autophagy within the neuron, and its function in cell survival. Indeed, Gigaxonin loss-of-function causes widespread neuronal death throughout the nervous system in giant axonal neuropathy patients^24^, and a progressive degeneration of GAN cortical neurons (our data). Additional studies will be needed to determine whether the autophagic impairment, which occurs prior to cell death, is the primary cause or is a contributing factor to neurodegeneration in GAN.

Neurodegeneration is one of the many different afflictions that autophagy impairment can cause to human. Indeed, autophagy is altered in a wide number of conditions, including cancer, myopathies, immune and neurodegenerative diseases^3,5^. While most of the mutated genes are not autophagic components per se, a growing body of evidence shows that they play additional roles in regulating autophagy^3,5^. Among them, *GAN* represents one of the few disease-associated genes, which encode for proteins that are either autophagic core components (WIPI4, ATG16L1) or their regulator (Gigaxonin), causing respectively encephalopathy^43^, Crohn’s disease^44^ and giant axonal neuropathy^24^. Our data compile to evidence that Gigaxonin acts by poly-ubiquitinating ATG16L1 with K48 chains, which leads to its degradation, but do not exclude other (non)ubiquitin-dependent control by Gigaxonin, which could regulate ATG16L1 trafficking within neurons. Altogether, our study further adds to the emerging concept of a cross-talk between the Ubiquitin Proteasome System and the autophagy pathway^45^. Our data pinpoints the first E3 ligase to date that controls the membrane elongation step^31^, hence unveiling a molecular switch to fine-tune the activation of autophagy.

## Methods

### Cell culture and transient transfection

COS-7 cells (clone CRL-1651 from ATCC) and HEK293T cells (ATCC CRL-11268) were maintained in Dulbecco's modification of Eagle medium (DMEM) medium (Thermo Fischer) containing 10% fetal bovine serum (Thermofisher) and 1% Penicilline/Streptomicine. Cells were tested negative for mycoplasma. Cortical neurons were obtained from E15.5 old embryos from the *GAN* ^*-/-*^ model^29^ and control littermates. Animals were treated in accordance with the European Union guide for the care and the use of animals in research (2010/63/UE). Briefly, brains cortices were dissected in HBSS (Thermofisher) supplemented with 0.44% glucose; enzymatically dissociated in HAMF10/0.025% trypsin (thermofisher); mechanically dissociated in neurobasal medium/2% fetal bovine serum, and concentrated after centrifugation at 470 × *g* on BSA cushions (Sigma). Neurons were re-suspended in Neurobasal Medium (Thermofisher), supplemented with 1% Sodium pyruvate (Thermofisher), 2% B27-Supplement (Thermofisher) and 1% Penicillin/Streptomycin (Thermofisher). Cells were plated on 14 mm-diameter coverslips (for immunostaining), or in 35 mm petri dish (for immunoblot), previously coated with 3 µg/ml of poly-D-ornithine (Sigma) and 2 µg/ml of laminin (Sigma), at a density of 1–2 x 10^4^ cells or 0.75–1 x 10^6^ cells, respectively. Transfection of plasmids was performed using Lipofectamin 2000 (Invitrogen) according to the manufacturer’s instructions. For induction of starvation-mediated autophagy, DMEM medium was replaced with EBSS (Thermofisher, SH30029.02) for 2 h or 6 h, as indicated. Lysosomal and proteasome inhibition was achieved by 200 nM Bafilomycin A1 (Sigma, B1793) and 20 µM MG132 (Tocris Bioscience) for 12 h.

### Autophagic measurements

Autophagy activity and flux was determined using complementary approaches: quantification of (i) LC3 lipidation, (ii) LC3 puncta formation, (iii) p62 aggregation, (iv) lysosomal fusion. For the quantification of LC3 lipidation, protein extracts were prepared as described in the western blot section. LC3-II and tubulin control were detected by rabbit anti-LC3B (1:1000, L7543, Sigma) and mouse anti-α tubulin (1:200, clone DM1α, Calbiochem), respectively. Quantification of LC3-II/tubulin intensity was performed by the Image-Lab software (http://www.bio-rad.com/fr-fr/product/image-lab-software) and the level of LC3 lipidation was determined by the relative LC3-II/tubulin ratio. LC3 flux was determined after comparison of the basal condition with serum deprivation condition (EBSS), with or without lysosome inhibitor (Bafilomycin A1). Autophagosome synthesis was measured as the differences of LC3 lipidation at two different times points in EBBS+Baf, at 2 h and 6 h. All values were expressed relatively to the LC3-II/tubulin ratio in wild-type neurons in basal condition (fixed to 1).

For the LC3 puncta formation, cortical neurons were fixed as described in the immunostaining section. LC3 fluorescence intensity of individual cell was quantified by ImageJ software (https://imagej.nih.gov/ij/).

P62 aggregation in primary neurons was visualised by immunofluorescence, using rabbit anti-p62 (1:1000, PM045 MBL) and mouse anti-MAP2 (1:1000, M4403, Sigma), in basal condition and EBSS, Baf and EBSS + Baf conditions. Lysosomal fusion was assessed by two independent analyses, by immunofluorescence on cortical neurons. The colocalisation of LC3 dots with lysosomes (lysotracker, 75 nM, L7528, Thermofisher) was quantified in individual neurons by the ImageJ software (https://imagej.nih.gov/ij/) with the Pearson coefficient. The fusion of p62 with lysosomes was assessed by immunofluorescence.

### Lentiviral infection

The lentiviral vector pLEX-FLAG-Gigaxonin, pLEX-GFP, p-VSVG and pAX2 were gifts from R.D. Goldman/P. Opal^46^. Lentiviral particles were produced after cotransfection of the pLEX-MCS-FLAG-Gigaxonin or pLEX-MCS-GFP plasmids, together with the helper plasmid p-VSVG and pAX2 into HEK293T cells. The supernatant was collected from the cells 2 days later and the virus was concentrated by ultracentrifugation. Primary cortical neurons from *GAN*^-/-^ mouse and control littermates were infected at 2 days in vitro (*div*) with mock-GFP and Flag-Gigaxonin lentivirus. Medium was changed 6–8 h after transduction and cells were examined at 4 *div*.

### Plasmids and siRNA

The human Cherry-ATG16L1 plasmid was generated by reverse transcription (SuperScript III Kit, Invitrogen) from HeLa cells mRNA. cDNA was amplified using primers flanked by ATTB1/ATTB2 sequences, and subcloned into gateway vectors. Flag-ATG16L1 mouse deletion constructs were generously provided by Pr Yoshimori^17^. The human Full-length and deletion Gigaxonin cDNAs were amplified from^47,48^ using ATTB1/ATTB2 primers. Final gateway plasmids were pcDNA-Cherry-N or pCi-3xFlag-N. For the Bimolecular Fluorescence Complementation (BiFC) assay, ATG16L1 and Gigaxonin cDNA were cloned in Gateway BiFC vectors, in fusion with the N-terminal region (YFPN: 1-154aa) and the C-terminal region (YFPC: 155-239aa) of the YFP protein, respectively. The primers used are the followings and are common across gateway vectors (ATTB sequences in lower cases):

ATG16L1 (Forward 5′ggggacaagtttgtacaaaaaagcaggcttcATGTCGTCGGGCCTCCGCGCC 3′, Reverse 5′ggggaccactttgtacaagaaagctgggttGTACTGTGCCCACAGCACAGC 3′); Full-length Gigaxonin (Forward 5′ ggggacaagtttgtacaaaaaagcaggcttcATGGCTGAGGGCAGTGCCGTG 3′, Reverse 5′ ggggaccactttgtacaagaaagctgggttAGGGGAATGAACACGAATACG 3′); ∆N Gigaxonin (Forward 5′ ggggacaagtttgtacaaaaaagcaggcttcGCACTACATTACTGCCTCCAT 3′, Reverse 5′ ggggaccactttgtacaagaaagctgggttAGGGGAATGAACACGAATACG 3′); ∆C Gigaxonin (Forward 5′ ggggacaagtttgtacaaaaaagcaggcttcATGGCTGAGGGCAGTGCCGTG 3′, Reverse 5′ ggggaccactttgtacaagaaagctgggttAGCTGACATAACATCCTTCAT 3′).

Silencing of Gigaxonin was performed using siRNAs (Dharmacon) of the following antisense sequences: siRNA-5′ AUAACAUAAAUACUGGCUC 3′ with the mismatch counterpart ms antisense sequence−5′ AUAAAAUAAAUACGGGCUC 3′.

### Western blotting and immunostaining

Cells were lysed in a solution containing 50 mM Tris pH 7.5, 150 mM NaCl, 1% Triton X-100, 5 mM EDTA and a cocktail of protease inhibitors^48^. Proteins were separated in 10–15% sodium dodecyl sulfate polyacrylamide gel electrophoresis (SDS-PAGE) gels, and transferred to nitro-cellulose membrane (hybond C-Extra, Amersham Biosciences). After blocking with 5% non-fat milk in PBS-0.05% Tween, membranes were incubated with primary and HRP-secondary antibodies and the signals detected by Chemiluminescent substrate HRP (Thermofisher, Millipore) using a conventional developer or a digital detection system (ChemiDoc, Biorad). Alternatively, fluorescent-labelled secondary antibodies were used and detection was performed thanks to the Odyssey® CLx imaging system (LI-COR). Uncropped scans of immunoblots are included as Supplementary Fig. 5. For immunofluorescence, cells were fixed with 4% Paraformaldehyde (PFA) for 15 min Blocking and permeabilisation were performed in PBS/0.1%Triton, 4% Bovine Serum Albumine (Sigma), 4% Donkey Serum (Sigma) for 1 h at room temperature. Subsequently primary antibodies, diluted in blocking buffer was incubated overnight at 4 °C. Following washes with PBS/0.1%Triton, secondary antibodies was incubated for 1 h at room temperature, and mounted in Mowiol solution (Sigma, 81381).

### Antibodies and reagents

Primary antibodies were from following sources: mouse anti-Cherry (1:1000, ab125096 Abcam), mouse anti-Flag (1:1000, F3165, Sigma), mouse FITC anti-Flag (1:1000, F4049, Sigma), mouse anti-GAPDH (1:2000, AM4300, Ambion), mouse anti-α tubulin (1:200, clone DM1α, Calbiochem), rabbit anti-HA (1:2000, H6908, Sigma), mouse anti-HA (1:1000, #26183, Thermoscientific), rabbit anti-doublecortin Dbx (1:1000, ab18723, Abcam), mouse anti-MAP2 (1:1000, M4403, Sigma), rabbit anti-ATG16L1 (1:1000, PM040, MBL), mouse anti-ATG5 (1:100 immunostaining, 1:1000 immunoblotting, M153-3, MBL), rabbit anti-LC3B (1:1000, L7543, Sigma), rabbit anti-p62 (1:1000, PM045, MBL), rabbit anti-Gigaxonin (1:1000, sab4200104, Sigma), mouse anti-Gigaxonin N12^48^ (1:150), rabbit anti-K48 chain specific (1:1000, Apu2, millipore), mouse anti-polyubiqutin chains (1:500, FK1, Enzo). For the immunoblotting, HRP-secondary antibodies were from the following sources: goat anti-rabbit (1:5000, #31460, Thermofisher), goat anti-mouse (1:5000, #31430, Thermofisher), rat anti-mouse IgG (1:1000, ab131368, Abcam). Fluorescent-labelled secondary antibodies used for immunoblotting were: donkey anti-mouse IRDye 800 CW (1:15000, #926–32212, Eurobio), donkey anti-rabbit IRDye 680 RD (1:15000, #926–68073, Eurobio). For immunofluorescence, Alexa 488, Alexa 594 and Alexa 647-conjugated secondary antibodies were from Jackson Labs. Lysotracker dye (Molecular probes, L7528, Thermofisher), used to to stain lysosome, was applied for 30 min at 50 nM concentration. Fluorescence pictures were taken with confocal laser scanning microscope model LSM700 (Carl Zeiss).

### Co-immunoprecipitation

Protein extracts were prepared from transiently transfected COS cells in the presence of the proteasome inhibitor MG132 (1748/5, Tocris). Epitope-tagged proteins were immunoprecipitated with anti-Flag and anti-Cherry antibodies as following. Negative controls correspond to Normal Mouse IgG (SC2025, Santa Cruz). Antibodies, linked with 50 µl Dynabeads Protein G (10004D, Thermofisher) (3 h, at room temperature in 500 µl PBS tween 0.02%), were incubated with 500 µg of proteins for 2 h at room temperature in 500 µl PBS tween 0.02%. Proteins-Antibody-bead complexes were washed with PBS tween 0.02%, re-suspended in Laemmli 1X solution and eluted at 70 °C for 10 min The totality of the sample was loaded on SDS-Page gel, while inputs correspond to 50 µg of initial protein lysates.

### Ubiquitination assay

In vivo ubiquitination assay of ATG16L1 was performed in COS cells, upon transfection of Cherry-ATG16L1 ± His-ubiquitin constructs and MG132 treatment. Whenever mentioned, siRNAs (siRNA) or mismatch siRNAs (ms) against the endogenous Gigaxonin were transfected the day before, using Dharmafect 2 reagent, and accordingly to the manufacturer’s instructions (Dharmacon). Protein extraction and ubiquitin pull down were performed under denaturing conditions, to examine only the covalent binding of ubiquitin onto ATG16L1. Briefly, cells were recovered, washed in PBS 1x, and subsequently divided for a direct lysis (i) in a 2x laemmli buffer for the input samples and (ii) in guanidium buffer for the nickel agarose pull down. (i) Input samples were further lysed mechanically through a 23G syringe, boiled at 95 °C and protein concentration was quantified using a BCA kit (Pierce), prior to the addition of 0.5 M ß-mercaptoethanol. (ii) Sample lysis and binding of ubiquitinated proteins was performed simultaneously in solution I (6 M guanidium-HCl, 0.1 M Na_2_HPO_4_/NaH_2_PO_4_, 0.01 M Tris-HCl, pH 8.0) supplemented with 5 mM imidazole and 10 mM ß-mercaptoethanol, and with 50 µl of Ni^2+^-NTA-agarose beads, with overnight agitation at 4 °C. Subsequently, different denaturing washes were performed with: solution I supplemented with 10 mM ß-mercaptoethanol and 0.1% Triton X-100, solution II (8 M urea, 0.1 M Na_2_HPO_4_/NaH_2_PO_4_, 0.01 M Tris-HCl, pH 8.0) supplemented with 10 mM ß-mercaptoethanol and 0.1% Triton X-100 and solution III (8 M urea, 0.1 M Na_2_HPO_4_/NaH_2_PO_4_, 0.01 M Tris-HCl, pH 6.3) supplemented with 10 mM ß-mercaptoethanol and 0.1% Triton X-100. Elution was performed under agitation for 20 min within 80 µl of 200 mM imidazole, 0.15 M Tris-HCl pH 6.7, 30% glycerol, 0.72 M ß-mercaptoethanol, 5% SDS. For each sample, equal quantity of the elution was analysed, according to the determination of protein concentration in the respective input samples.

### Proximity ligation assay

Proximity ligation assay was performed according to the manufacturer’s instructions (Duolink In Situ-Fluorescence, Sigma). COS cells were transiently transfected with different combinations of plasmids: Ha-ubiquitin with ± Cherry-ATG16L1 and ± Flag-Gigaxonin. Fixation, blocking, and primary antibody incubation was performed as described in the immunofluorescence section. The PLA probes α-mouse minus and α-rabbit plus were diluted and mixed in ratio 1:5 (except for two negative controls where only one probe was added) in the Duolink in Situ Buffer for 20 min at room temperature, and subsequently applied to the cells for 1 h at 37 °C. After washing with the PLA wash buffer, ligation between the two PLA probes was achieved in the Ligation-Ligase solution, applied for 30 min at 37 °C. The phase of amplification was performed using the Amplification-Polymerase solution green for 100 min at 37 °C.

### Statistics analysis

The statistical significance of the difference between experimental groups was determined by the two-way ANOVA test (two quantitative variables: genotype and treatment/time; one qualitative factor) with Bonferroni post-hoc test (Figs. 5b and 7b) or by the two-tailed Mann–Whitney test (Fig. 5d, Supplementary Fig. 3b), after an assessment of the normality of distribution of the data. Accordingly, either means or medians are represented in the figures, and individual values are shown. The sample sizes (*n*) are indicated in each figure. Individual measures were normalised to internal controls (tubulin for Fig. 5a, DAPI for Fig. 7a) or background intensity (Fig. 5d and Supplementary Fig. 3b). In addition, GAN values were normalised to control values in every quantification. As presented in the figures, the differences between experimental groups are significant for *P < 0.05, **P < 0.01, ***P < 0.001 and ****P < 0.0001.

## Supplementary information

Supplementary Information

## Data Availability

The data that support the findings of this study are available from the corresponding author upon reasonable request.
