# Supplementary material for: Gigaxonin E3 ligase governs ATG16L1 turnover to control autophagosome production
Source: Nat Commun. 2019 Feb 15;10:780. doi: 10.1038/s41467-019-08331-w (PMC6377711; doi:10.1038/s41467-019-08331-w)

Supplementary Information

Gigaxonin E3 ligase governs ATG16L1 turn over
to control autophagosome production

Scrivo et al.

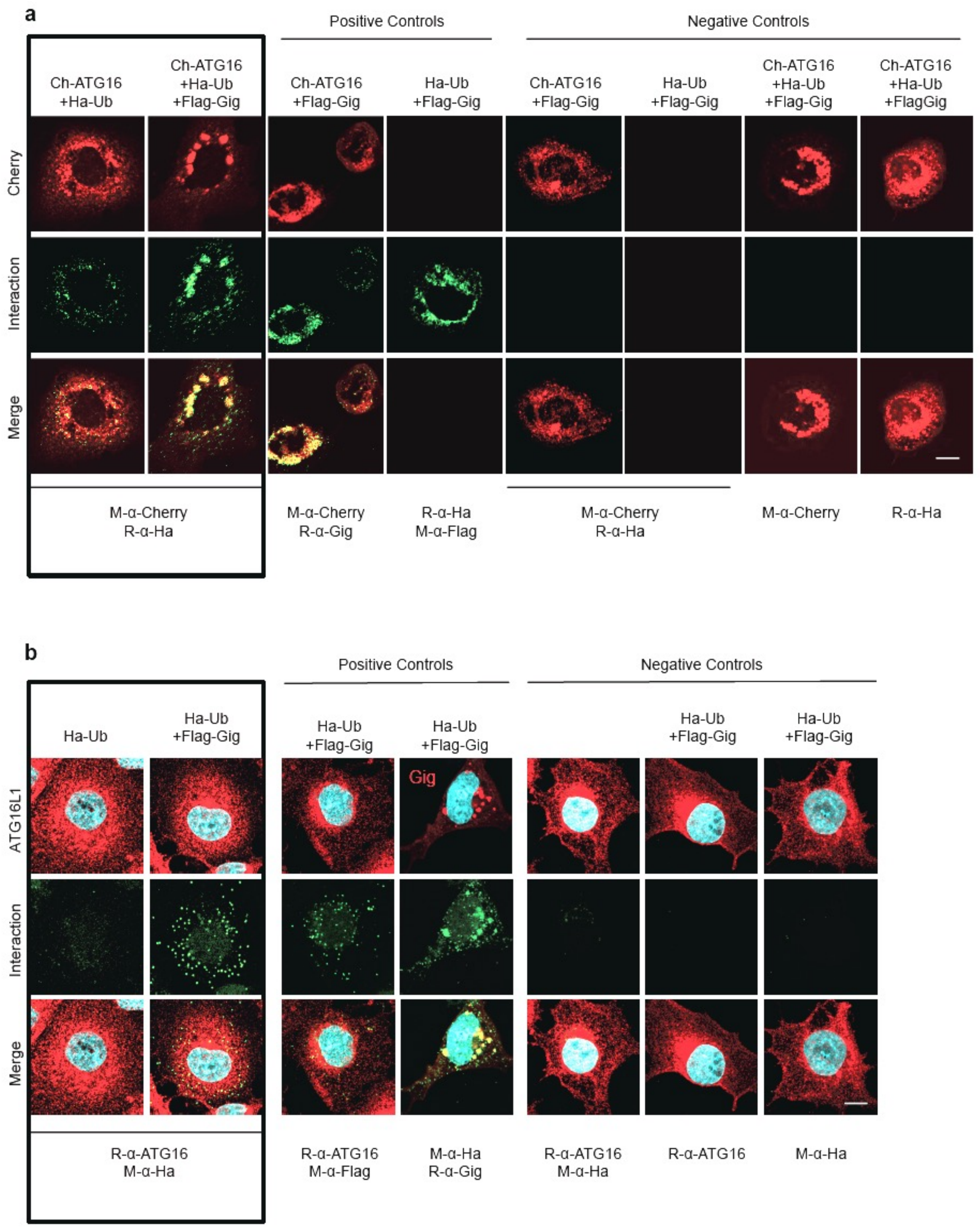

Supplementary Figure 1 Specificity of the reactions of the Proximity Ligation Assay as presented in Fig. 3, to show the increased colocalisation of ubiquitin with the exogenous (**a**) and endogenous (**b**) ATG16L1 protein by the Gigaxonin-E3 ligase. **a-b** The positive controls validate the ability of each of the primary antibodies to amplify the PLA reaction. The negative controls show the absence of aspecific staining by i) combining both primary antibodies in condition with one of the transfected constructs missing, and ii) using only one of the primary antibodies with all constructs. Please note that the positive controls confirmed the interaction of Gigaxonin with ectopic ATG16L1 (**a**) and the endogenous ATG16L1 (**b**). In all experiments, red channels correspond to ATG16L1 (either endogenous protein in **b**, or overexpressed protein in **a**), with the exception of the picture were Gig is mentioned. Scale bar: 10µm

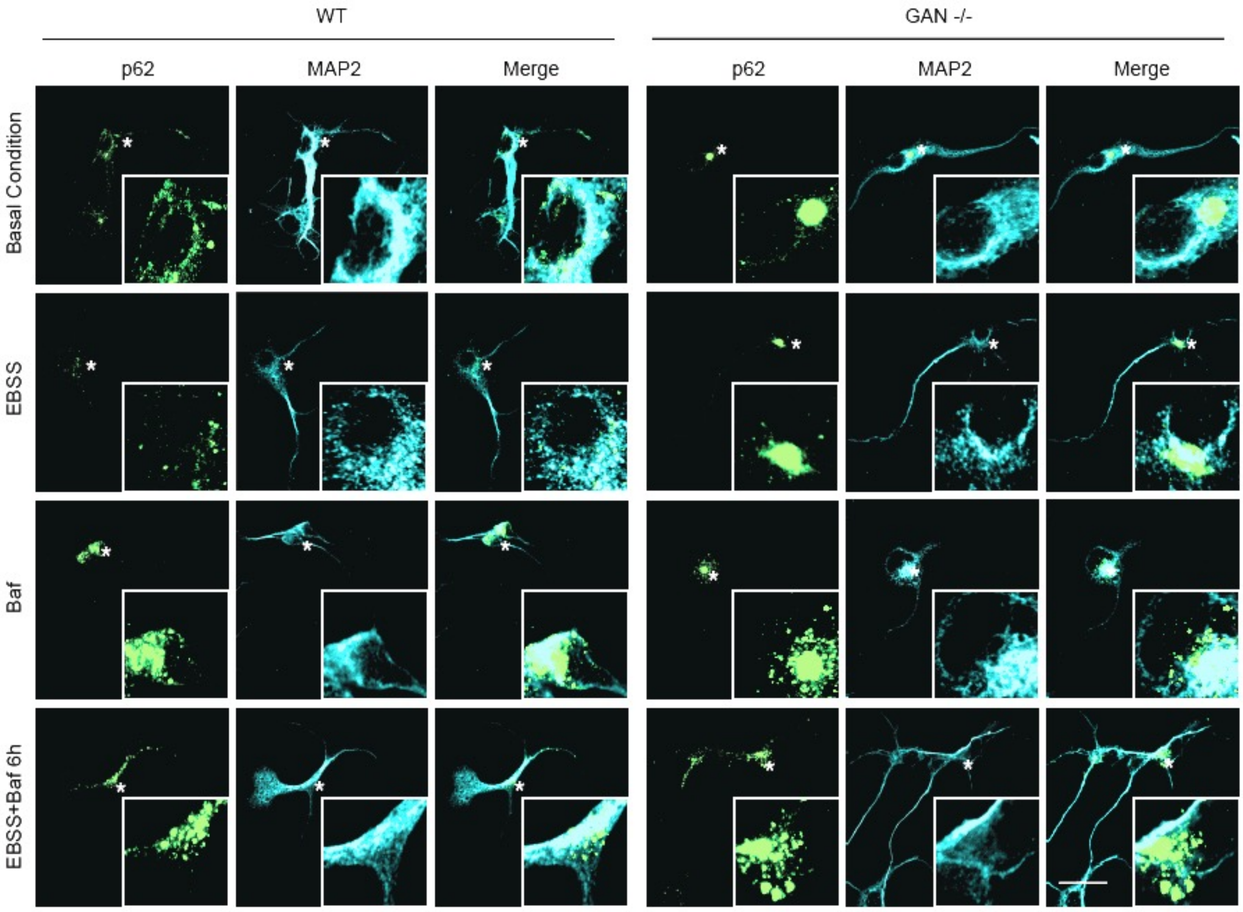

Supplementary Figure 2 Gigaxonin repression induces the aggregation of the autophagy receptor p62. Immunolabelling of control cortical neurons with p62 to show its constitutive degradation in basal and EBBS conditions, and its accumulation upon inhibition of autophagy degradation (Baf and EBSS+Baf6h). Perinuclear aggregates of p62 were detected in all conditions in *GAN*$^{-/-}$ neurons, regardless of autophagy degradation. Scale bar: 20µm

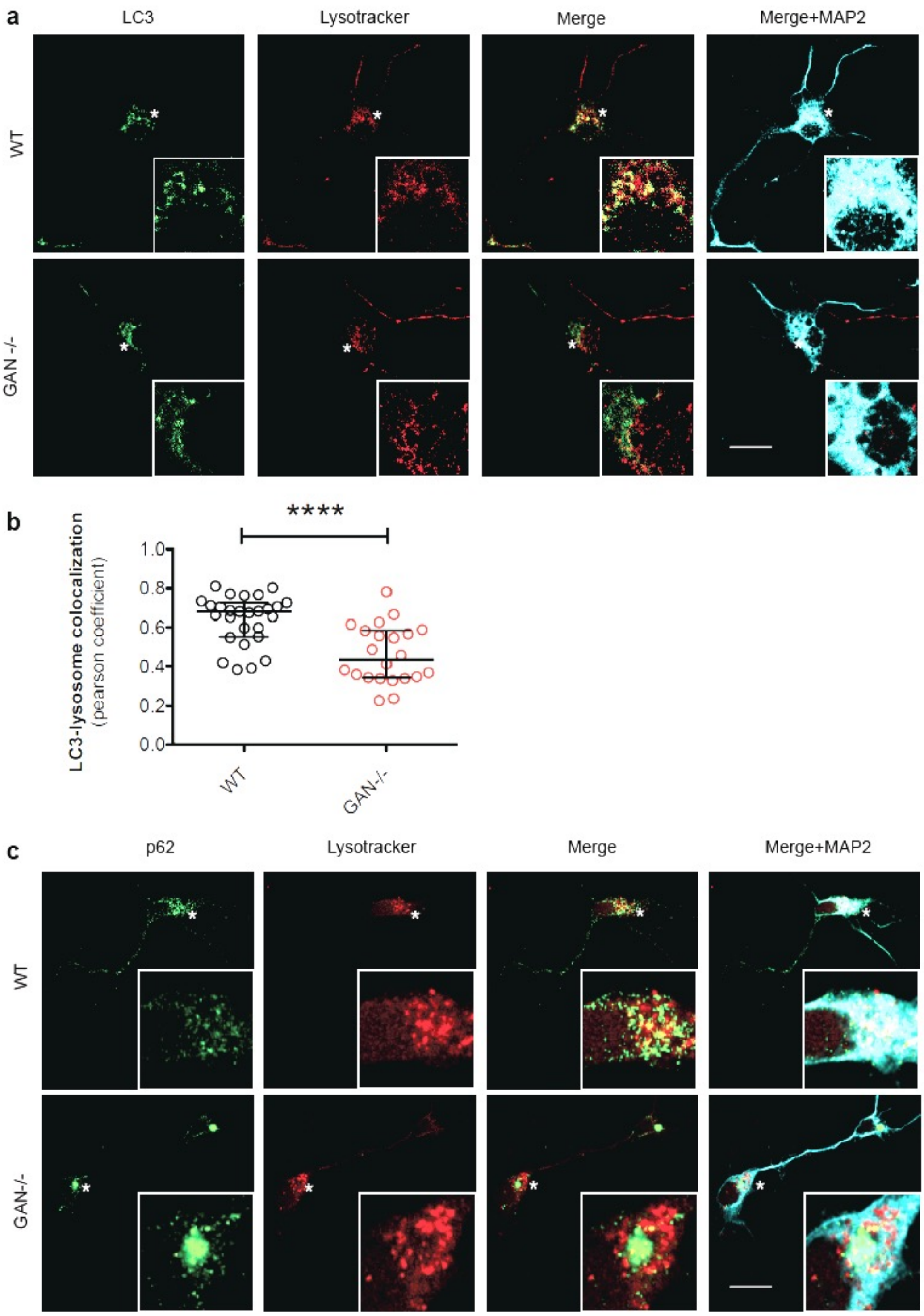

Supplementary Figure 3 GAN $^{-/-}$ neurons are defective in autophagosome-lysosome fusion. Co-staining of LC3 **(a)** or p62 **(c)** with lysotracker evidenced decreased fusion between autophagic structures and lysosomes in *GAN*$^{-/-}$ neurons. **(b)** The decrease in the colocalisation of LC3 with the lysotracker was determined by the Pearson Coefficient. N=27 for wild type cells represented in black circles, and n=22 for mutant cells represented in red circles, from three independent experiments; individual measures and medians with interquartile range are represented; statistical significance was obtained with a ****P<0.0001 value with the two-tailed Mann Whitney test. Scale bar: 20µm

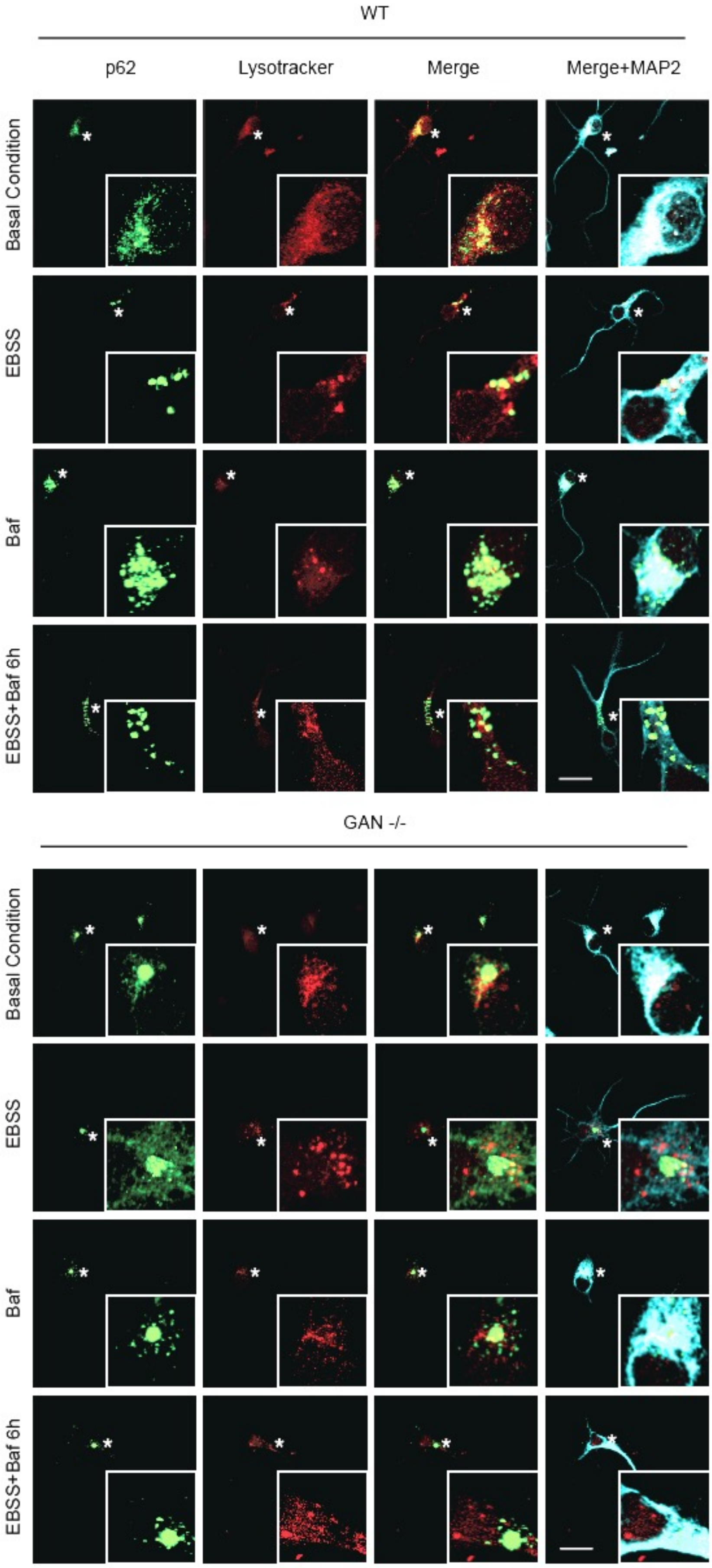

Supplementary Figure 4 Autophagy induction does not promote autophagosome maturation in $GAN^{-/-}$ neurons. Immunofluoresence of p62 and lysotracker was performed in various conditions in wild type cells, to illustrate the increased and decreased autophagosome-lysosome fusion upon, respectively, serum deprivation (EBSS) and inhibition of the fusion (Baf and EBSS+Baf6h). Note that p62 content increases in all treatments, as a result of increased autophagosome production (EBSS) and/or accumulation (Baf, EBSS+Baf6h), but does not generate aggregates. P62 was equally excluded from lysosomal compartments in all conditions in $GAN^{-/-}$ neurons, demonstrating their deficiency in targeting autophagic structures to lysosomal degradation. Scale bar: 20μm

Supplementary Figure 5

Figure 1 panel e

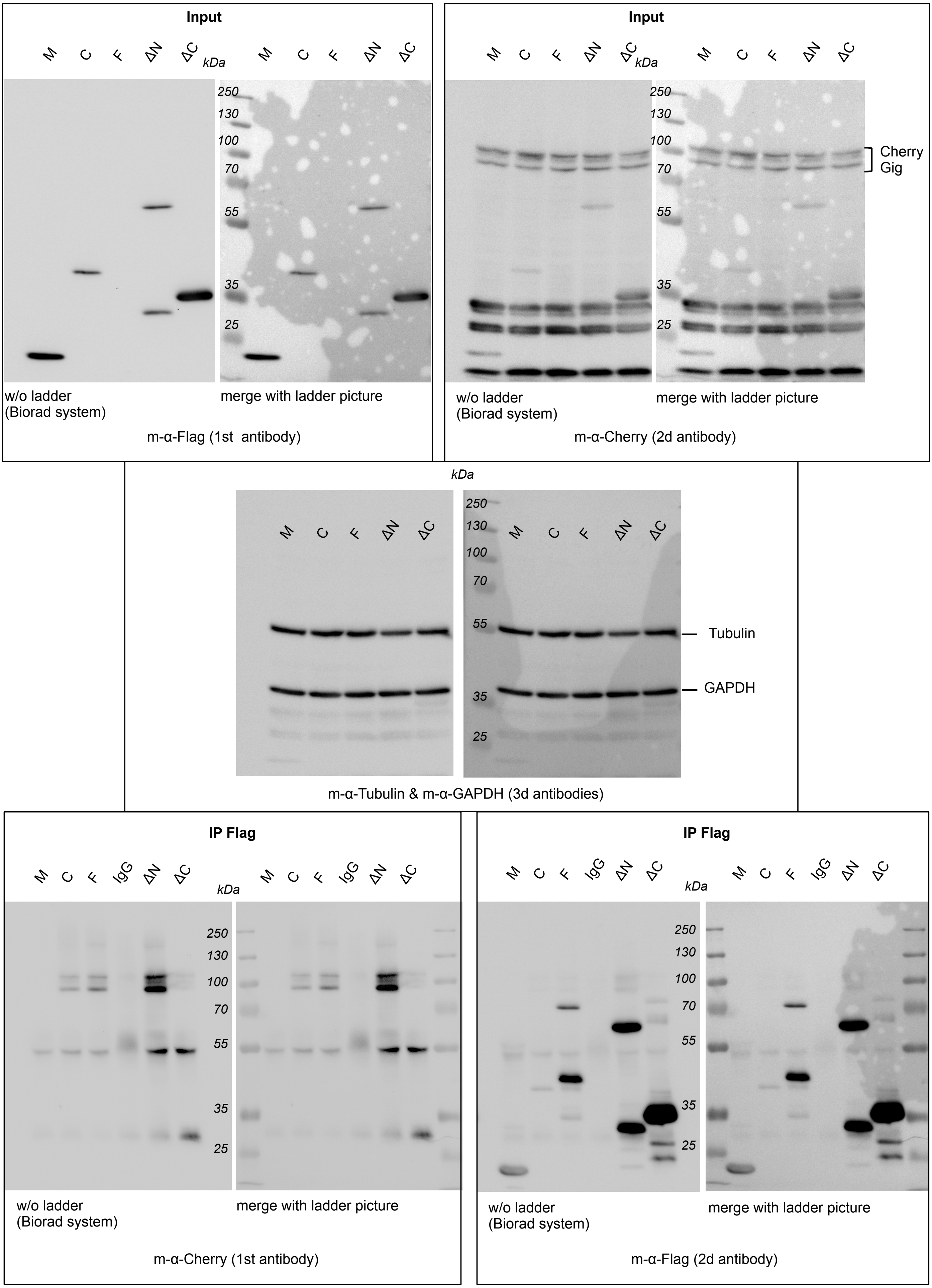

Figure 1 panel g

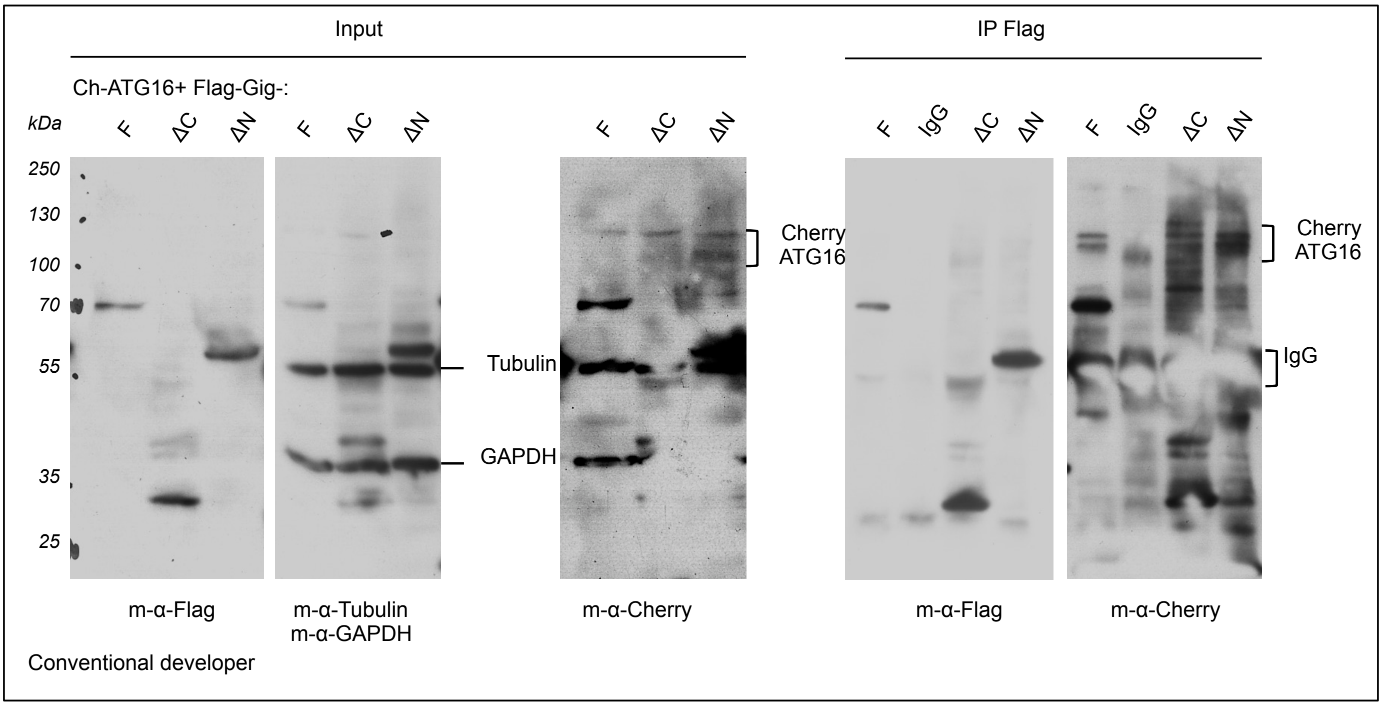

Figure 5 panel a

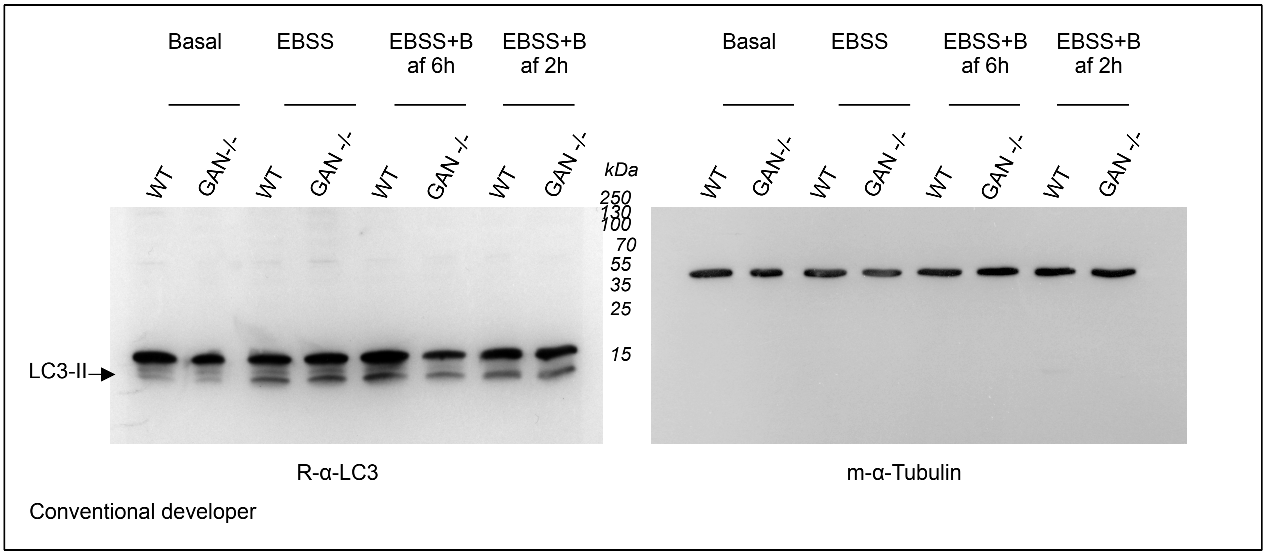

Figure 6 panel c

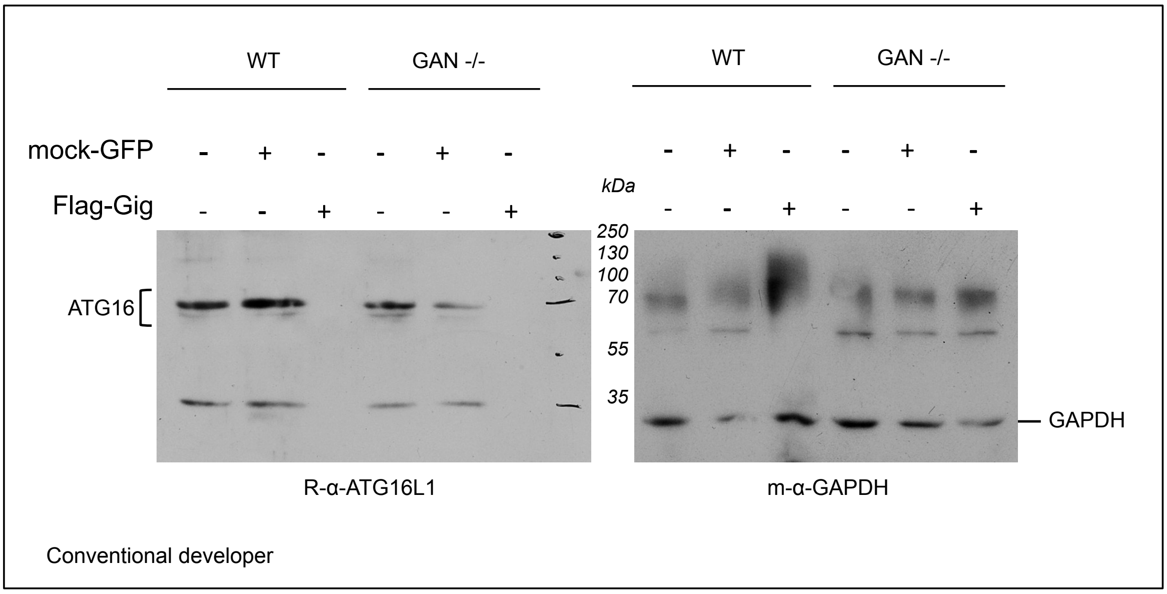

Figure 2 panel b

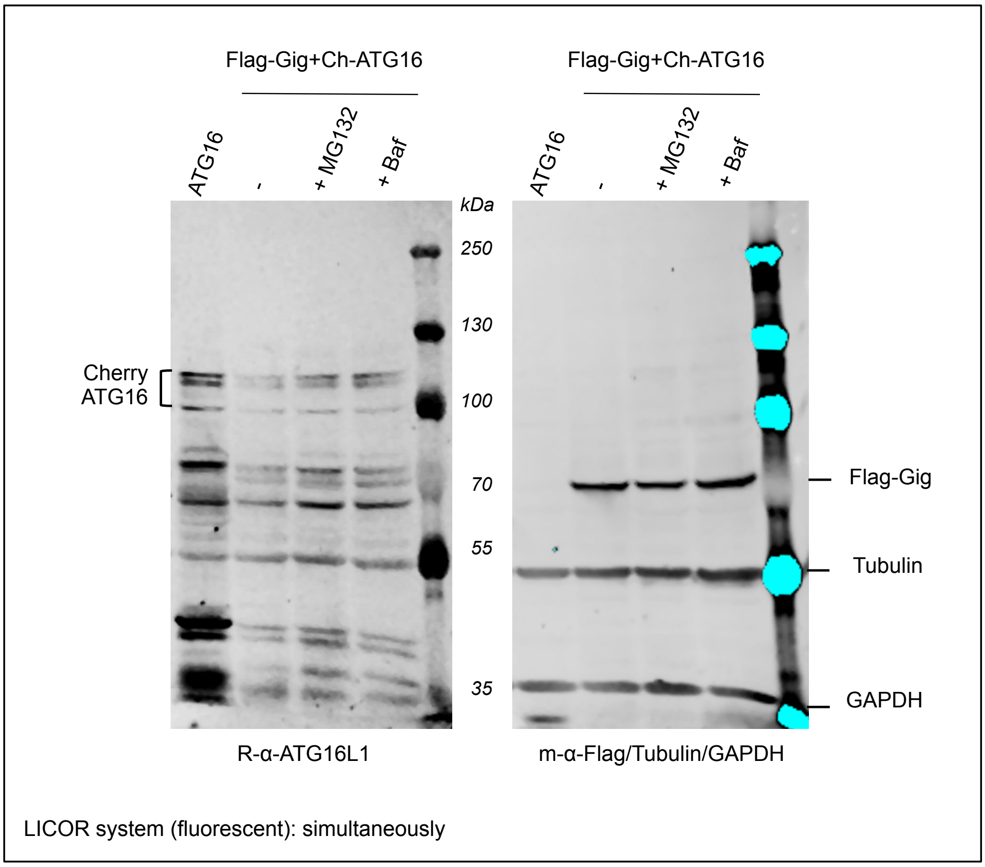

Figure 2 panel d

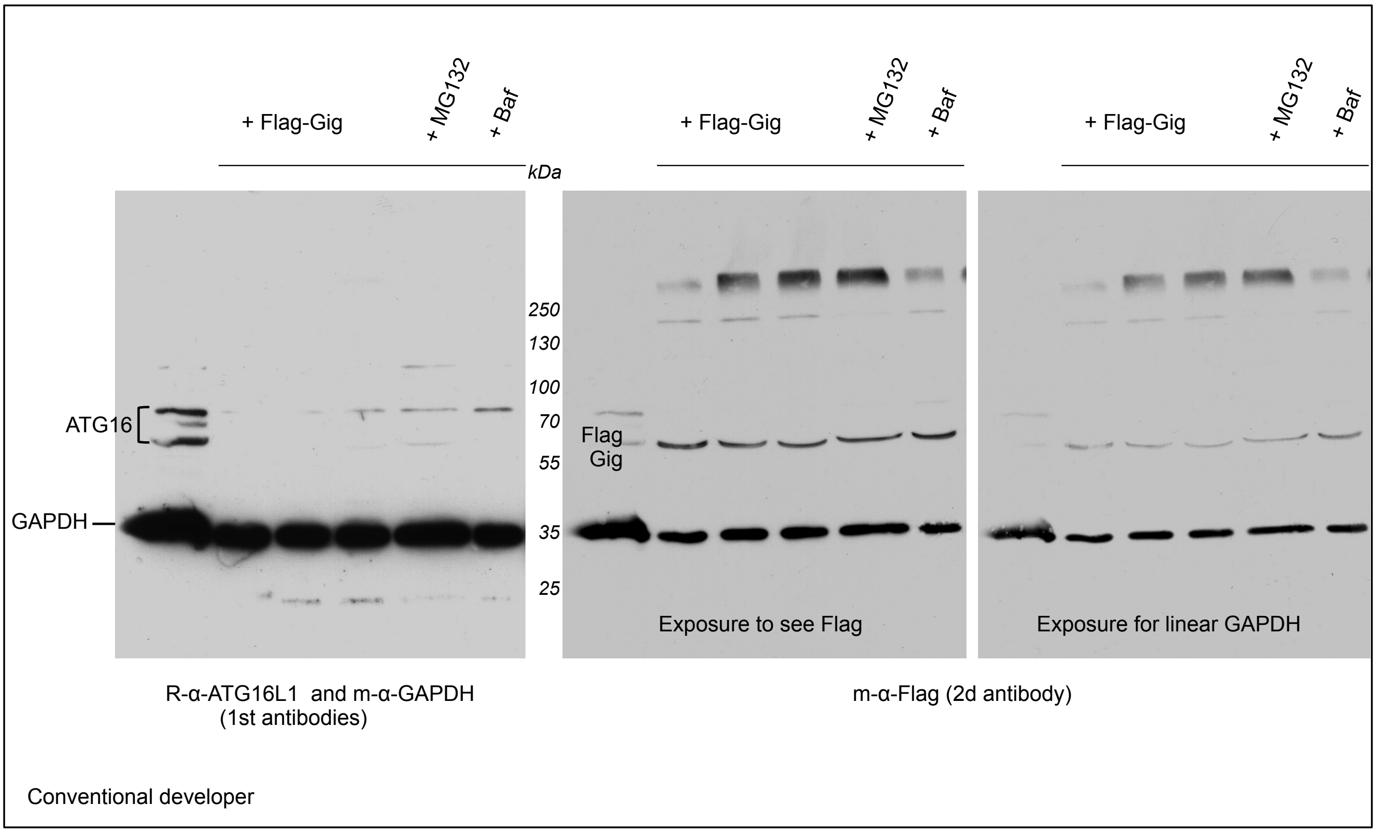

Figure 3 panel a

Input

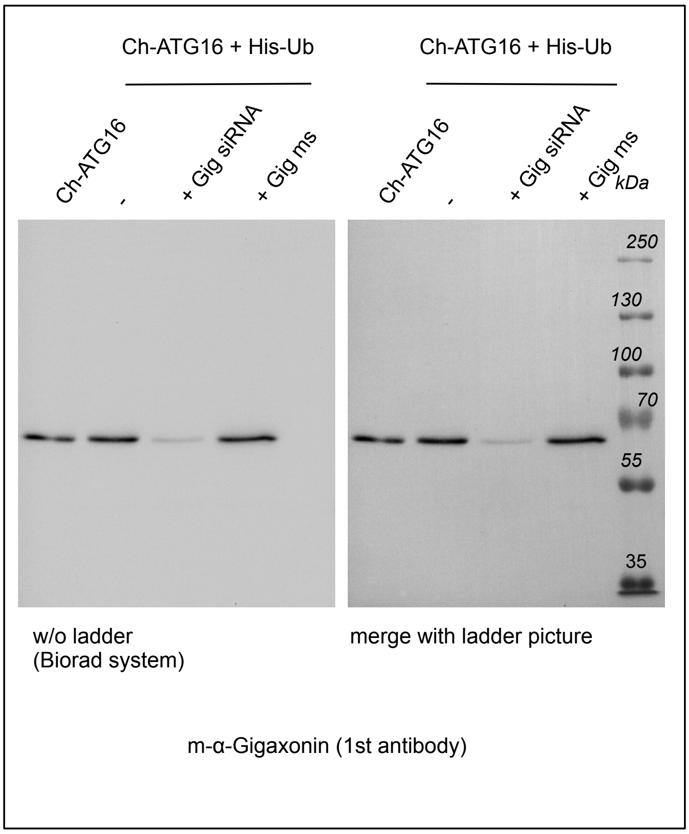

Input

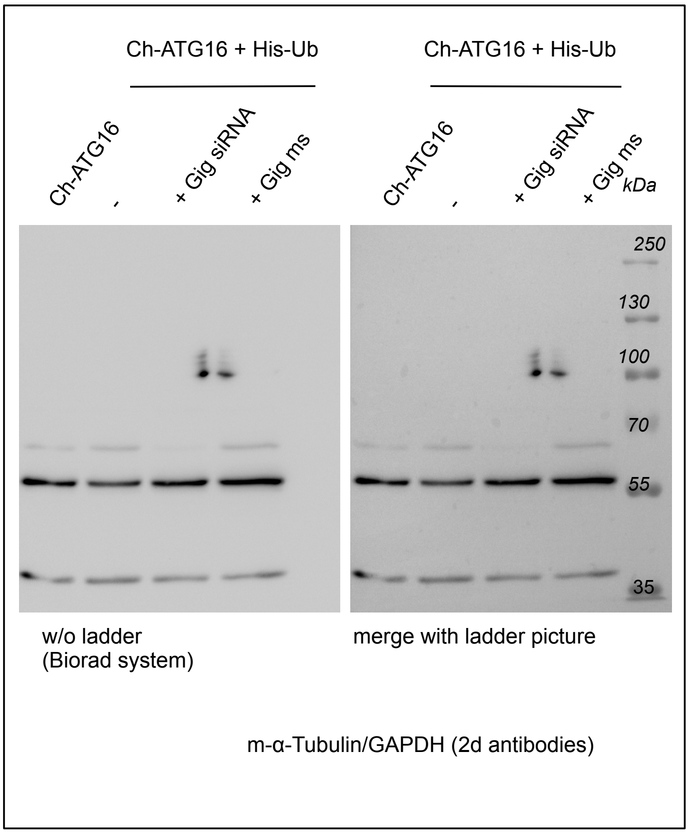

Input

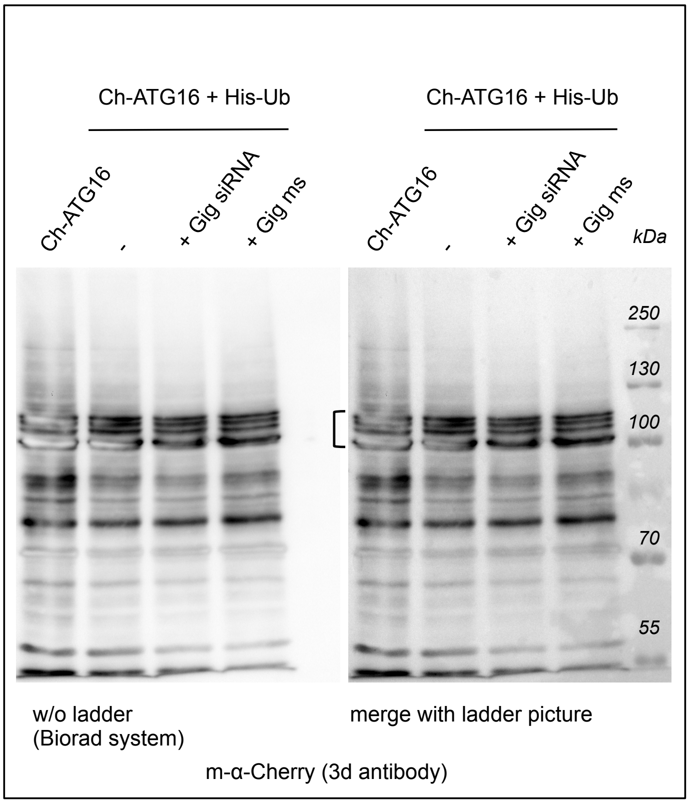

His pull-down

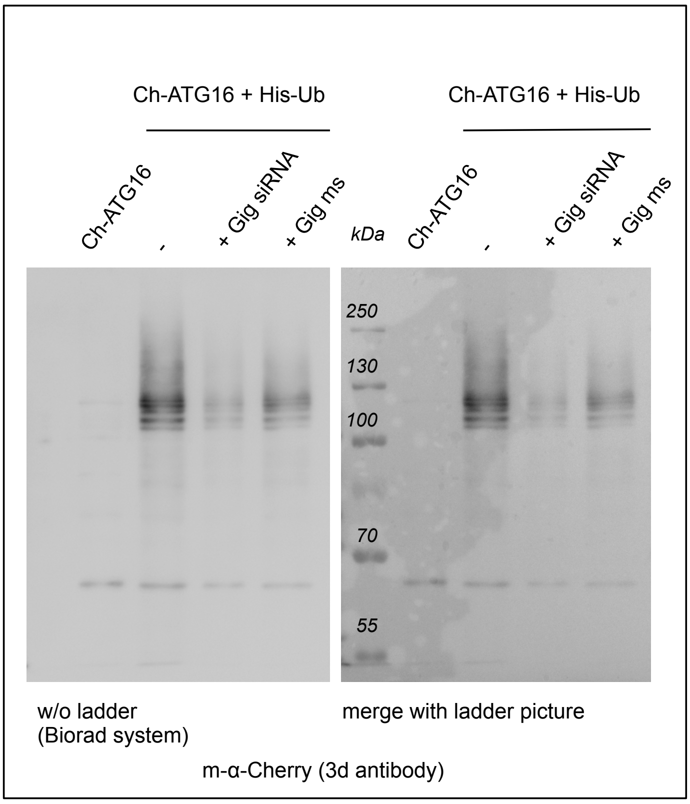

Supplement: Supplementary file 1 — Supplementary Information [file 41467_2019_8331_MOESM1_ESM.pdf]
